# Menstrual health among young adults in Latin America and the Caribbean: A scoping review and evidence-gap map

**DOI:** 10.1177/17455057251379612

**Published:** 2025-10-31

**Authors:** Lisa Irene Jones, Michelle A. Krahe, Nicola Rahman, Neil Harris, Nicola Wiseman, Gabriela Bustamante

**Affiliations:** 1 School of Medicine and Dentistry, Griffith University, Gold Coast, Queensland, Australia; 2 Instituto de Medicina Social & Desafíos Globales, School of Public Health, Universidad San Francisco de Quito, Quito, Pichincha, Ecuador; 3 College of Medicine and Dentistry, James Cook University, Smithfield, Queensland, Australia

**Keywords:** dysmenorrhea, Latin America, menstruation, menstrual hygiene products, students, young adult

## Abstract

Menstrual health (MH) research has expanded in recent years, including studies throughout the reproductive life course. However, the experiences of young adults in Latin America and the Caribbean (LAC) remain comparatively underexplored, despite the importance of this life stage. The primary aim of this scoping review was to summarize and synthesize the literature on MH among young adults in the LAC region. Primary studies were included if they explored any aspect of MH among young adults aged 18–24 in university, health service, or community settings within LAC. Studies conducted in secondary school settings or involving specialized populations (e.g., elite athletes, incarcerated individuals) were excluded. Six electronic databases were searched for studies published between January 1, 1980 and October 23, 2024. Data were extracted and synthesized narratively, with key study characteristics summarized in tables. An interactive online evidence-gap map was developed to visualize geographic and thematic representation across the region. A total of 42 studies met the inclusion criteria, with most originating from Brazil (*n* = 21, 50%) and Mexico (*n* = 14, 33%). Studies were predominantly conducted in urban locations (*n* = 29, 69%), and all participants were described as female or women. Common MH experiences reported included menstrual cycle and bleeding characteristics (*n* = 16, 38%), dysmenorrhea (*n* = 13, 31%), and premenstrual syndrome (*n* = 10, 24%). Some studies indicated a negative impact of MH on academic participation (*n* = 5, 12%) and daily life activities (*n* = 16, 38%). The review identified significant geographic gaps, with only 21% (7/33) of LAC countries represented. This review highlights significant gaps in MH research among young adults in LAC, especially in rural areas and gender-diverse populations. There is a critical need for inclusive, region-specific research, initiatives, interventions and policies to enhance health, education and economic outcomes.

## Introduction

Approximately 300 million individuals presumed female at birth (PFAB), including cisgender women and girls, transgender, gender-diverse, and intersex people, menstruate each day, making menstrual health (MH) a critical determinant of physical, mental, and social well-being.^1,2^ MH encompasses access to accurate information, menstrual products, water, sanitation, and hygiene (WASH) infrastructure, supportive care, and a stigma-free environment that allows full participation in daily life.^3^ Earlier MH research focused largely on adolescents in school settings, particularly in sub-Saharan Africa and South Asia.^4–9^ More recent scholarship has responded to calls for expansion to older age groups and broader geographic regions, reflecting a growing recognition of the need for a more inclusive understanding of menstrual experiences across the life course.^10,11^

Young adults aged 18–24 have emerged as an important population in this shift. This life stage is often marked by key transitions, such as entering higher education, joining the workforce, or becoming more independent, which can shape and complicate MH needs.^12^ For university students in particular, MH challenges such as pain, stigma, or inadequate support can hinder concentration, class attendance, and academic performance.^13,14^ This is significant as higher educational attainment in this age group is strongly associated with long-term health, social, and economic outcomes.^15–17^ A recent United Kingdom-based study found that gynecologic pain, including dysmenorrhea, impacted the educational engagement of predominantly young adults. Many participants reported difficulty accessing healthcare and navigating institutional support systems, citing the normalization of symptoms and dismissive provider interactions.^18^

While university students have increasingly become the focus of global MH research, important knowledge gaps remain, especially regarding how young adults experience menstruation in different cultural and regional contexts. It is important to note that while many young adults are enrolled in higher education, this population also includes those engaged in employment or other community settings. As such, understanding MH in this group requires attention not only to academic environments but also to broader aspects of daily life. Systematic reviews have highlighted the global burden of primary dysmenorrhea (PD) among students, estimating prevalence as high as 74.9% among PFAB university attendees.^13,14^ Dysmenorrhea, more generally, is associated not only with disruptions to academic performance, such as attendance and classroom concentration, but also with reduced participation in exercise, social interaction, and general well-being.^14,19–23^

However, these reviews have included very limited research from the Latin America and the Caribbean (LAC) region, with just one study each from Mexico represented in both student-focused reviews.^24,25^ Similarly, a broader review of adolescent MH across 45 countries included only five studies (11%) from LAC.^26^ Although MH research in the region does exist, for example, a recent study among Mexican PFAB university students found a high prevalence of PD (78.9%) and reported that symptom severity significantly interfered with academic performance,^27^ much of the available literature remains scattered and underrepresented in global syntheses.

Given that LAC comprises 33 countries with diverse social, economic, and cultural contexts,^28^ region-specific insights are essential for understanding how MH affects young adults’ academic engagement, social participation, and daily life. The LAC region faces persistent challenges in menstrual hygiene management, including limited access to WASH infrastructure and menstrual products, as well as insufficient MH education, which are essential factors in MH.^29^ Moreover, public health systems in several LAC countries face structural limitations that may restrict access to menstrual-related healthcare, particularly for youth and marginalized populations.^30–32^ While MH research in the region is growing, a comprehensive regional overview among the young adult population could support future practice, policy, and research efforts. The primary aim of this scoping review is to address this gap by summarizing and synthesizing available literature on MH among young adults PFAB aged 18–24 in the LAC region. Specifically, the review is guided by the following objectives:

1. To explore the reported experiences of MH among young adults in LAC,
2. To examine how these experiences influence participation in academic and daily life.

## Methods

This review followed the Arksey and O’Malley^33^ framework and is reported in accordance with the 2018 Preferred Reporting Items for Systematic Reviews and Meta-Analyses extension for Scoping Reviews criteria.^34^ The protocol was registered with the Open Science Framework https://doi.org/10.17605/OSF.IO/4YU35.

### Search strategy

A systematic search was conducted on March 23, 2023, using the population, concept, and context framework to structure the main concepts guiding the review aim, objectives, and eligibility criteria.^35^ The search was updated on October 23, 2024. Our *population* was young adults PFAB aged 18–24 years old, the *concept* involved MH experiences, emphasizing the lived experiences of menstruation, such as menstrual practices, attitudes, and challenges like PD and premenstrual syndrome (PMS) faced during the cycle, and the *context* were studies from LAC, including the Caribbean, Mexico, Central and South America. Databases searched included Medline via Ovid, ERIC via ProQuest, Latin America & Iberia Database via ProQuest, Psycinfo via Ovid, Web of Science, and Scopus, with search strategies adapted per database. The search strategy was externally reviewed by a senior health librarian for accuracy. Full search strategies are available in Supplemental Materials 1 and 2.

### Eligibility criteria

Detailed inclusion and exclusion criteria are available in Supplemental Material 3, with key areas expanded upon below.

#### Inclusion criteria

The search included studies published between January 1, 1980, and October 23, 2024, starting from the publication of the Menstrual Attitude Questionnaire in 1980.^36^ Although the Menstrual Distress Questionnaire (MDQ) was developed earlier in 1968,^37^ focusing on studies from 1980 onwards reflects contemporary practices and accounts for advancements in the field, including tools such as the Beliefs about and attitudes toward Menstruation Questionnaire (BATM)^38^ and the Premenstrual Symptoms Screening Tool (PSST).^39^ Studies published in English, Spanish, Portuguese, and French were eligible for inclusion to align with the linguistic diversity of the LAC region. Dutch and officially recognized Indigenous languages were excluded from full review due to the authorship team’s language limitations, though no studies in these languages appeared in our search.

Primary studies were included if they examined any aspect of MH among young adults PFAB aged 18–24 in university or non-university contexts, such as health services or community settings. Studies covering broader age ranges were included if they involved participants aged 18–24. The term *community* broadly encompasses workplaces, households, and other environments where specific settings were not identified, reflecting young adults’ engagement in diverse activities.^40,41^ MH experiences did not need to be the primary study outcome. Eligible studies had to be LAC-based, as defined by the World Bank Group,^28^ and could include qualitative and/or quantitative designs.

#### Exclusion criteria

We excluded validation-only studies, secondary studies, and research limited to abstracts (e.g., conference abstracts). Research reporting MH experiences from partners or physicians (e.g., gynecologists) was omitted to prioritize first-hand accounts. Experimental intervention studies and those focusing on menarche experiences were also excluded.

Studies conducted in secondary school settings were excluded, even if they included individuals PFAB aged 18–24, to focus on young adults in post-school environments such as universities and community settings. Specialized populations, such as airline staff, incarcerated individuals, detainees, and refugees, were excluded due to their context-specific needs. Migrant-focused studies were reviewed for potential inclusion given significant migration patterns in LAC, particularly among Venezuelans in Brazil, Chile, Colombia, Ecuador, and Peru,^42,43^ although migrants in informal settlements or in the process of traveling across borders were excluded. Studies on individuals PFAB with diagnosed affective disorders or disabilities were also excluded to account for their unique experiences.^44^ Studies on athletes were excluded due to their unique physical and training demands, which may not reflect the general young adult population. Similarly, research focusing exclusively on smaller minority groups or distinct indigenous populations was excluded to maintain broader applicability and comparability of findings across the LAC region.

Studies on eumenorrhea (normal menstrual bleeding every 21–35 days) were included to assess typical MH experiences. However, studies solely focused on conditions such as endometriosis, polycystic ovarian syndrome, chronic pelvic pain (CPP), moderate-severe PMS or premenstrual dysphoric disorder (PMDD), abnormal uterine bleeding, menorrhagia (heavy bleeding or bleeding for 7+ days), amenorrhea, or irregular, short, or long cycles were excluded, as these conditions affect less than 30% of the global population PFAB.^45–53^ This threshold was set to maintain a focused scope on the most common MH conditions, which are more likely to have widespread implications for academic and daily life in this population. Studies on PMS (mild-moderate) and PD were included due to their high prevalence, with 80% of reproductive-age individuals PFAB experiencing PMS symptoms and 50%–90% reporting PD.^54–57^ While studies focused on amenorrhea-related experiences were excluded, those exploring attitudes toward menstruation, including views on contraceptive-induced amenorrhea, were assessed for potential inclusion due to their broader relevance to menstrual attitudes. Studies focusing solely on sexual function or sleep quality were excluded unless linked to quality of life or other daily life outcomes, as the menstrual cycle’s influence on these areas^58,59^ was outside the review’s scope.

### Data extraction

One author (L.I.J.) initially removed duplicate records using Bramer et al.’s^60^ method by Gore,^61^ a widely used approach for systematic and thorough deduplication in EndNote20. Two authors (L.I.J. and N.R.) then independently screened titles and abstracts in Endnote20, resolving discrepancies through discussion. During the full-text review, both authors refined and finalized the inclusion and exclusion criteria. The updated search was managed through Covidence software,^62^ where duplicates were automatically removed, and L.I.J. screened titles and abstracts. Decisions during both screening and full-text review were discussed collaboratively with the authors.

A data charting table was developed following an adapted Arksey and O’Malley^33^ approach to systematically record key variables: author(s), year, study location (including remoteness), setting, sample size, participant descriptor (reported participant identity), age range (mean and standard deviation (SD)), study design, focus of MH experience, and its impact on academic and/or daily life. *Academic* impacts included absenteeism and effects on performance, concentration, task completion, and productivity. *Daily life* impacts encompass partner and family relationships, domestic, professional, functional, social activities, exercise/sports, quality of life, and general daily functioning. Missing data were requested from authors twice over 4 weeks via email (if contact information was available); unresponsive or unavailable data were classified as unknown.

### Data analysis

Findings are presented narratively and supplemented by a data charting table summarizing key study characteristics. The MH experience focus was classified based on the overall themes reported in the studies: menstrual cycle and bleeding characteristics, dysmenorrhea, management of menstrual-related symptoms, PMS, attitudes toward menstruation, menstrual practices, and unclassified menstrual-related symptoms. We specifically note dysmenorrhea rather than PD, acknowledging that these are distinct concepts; however, for this review, studies referring to either were categorized under the broader category of dysmenorrhea.

To visualize key findings, two evidence-gap maps were created. The first is a heatmap table illustrating the frequency and percentage of studies addressing key study characteristics. Varying shades of green indicate study concentration, with darker shades representing a higher number of studies in that area. The second map displays all included studies according to their MH focus area and whether they assessed impacts on academic or daily life participation. An interactive online version of this evidence-gap map was also developed using Kumu,^63^ allowing readers to filter studies by remoteness, country, and setting, and to explore study details by clicking on nodes.

## Results

**Figure 1.**
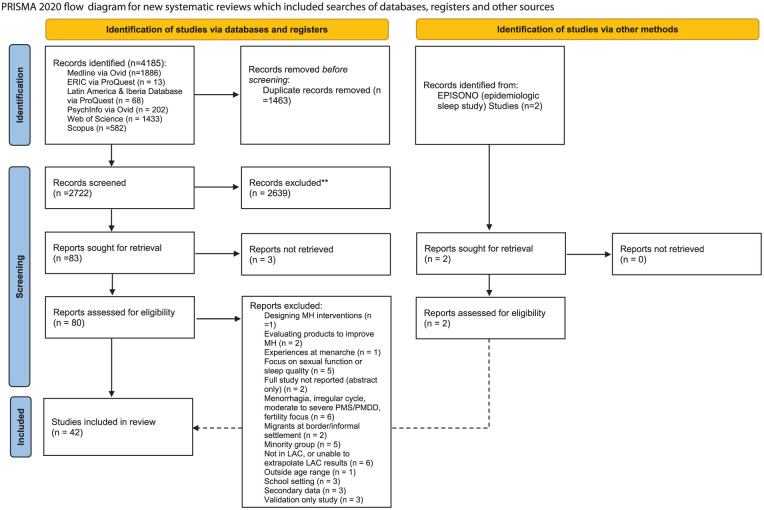
PRISMA flow diagram. EPISONO: São Paulo Epidemiologic Sleep Study; MH: menstrual health; PMS: premenstrual syndrome; PMDD: premenstrual dysphoric disorder; LAC: Latin America and the Caribbean; PRISMA: Preferred Reporting Items for Systematic Reviews and Meta-Analyses.

Four thousand eighty-five records were identified. After removing duplicates (*n* = 1463), 2722 records were screened by title and abstract, resulting in 83 reports eligible for full-text retrieval. Eighty were assessed in full text for eligibility; three reports could not be retrieved. Forty studies were included. One study utilized the 2007 São Paulo Epidemiologic Sleep Study database,^64^ prompting further searches of related MH studies, which revealed two additional studies for inclusion. In total, 42 studies were included in this review (see Figure 1).

### Study characteristics

**Figure 2.**
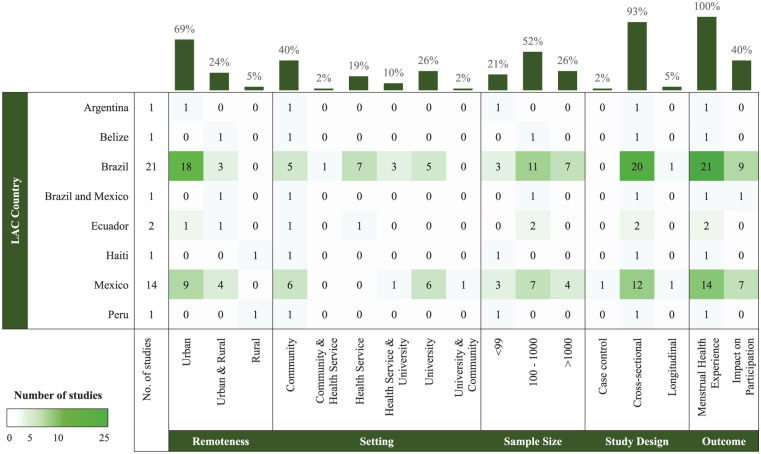
Evidence heatmap showing characteristics of included studies (*n* = 42), distributed by LAC country. Values within boxes indicate the number of studies, and bars show the percentage representation of each study characteristic. LAC: Latin America and the Caribbean.

Most studies included in this review used cross-sectional study designs (*n* = 39, 93%), with quantitative methods (*n* = 40, 95%) (Table 1 and Figure 2). Twenty-one studies (50%) originated from Brazil, 14 (33%) from Mexico, 2 from Ecuador, and 1 each from Argentina, Belize, Haiti, Peru, and 1 study that combined data from Brazil and Mexico. Studies conducted in urban areas were predominant, accounting for 69% (*n* = 29), followed by a mix of urban and rural (*n* = 10, 24%) and solely rural (*n* = 2, 5%). Studies recruited young people in university (*n* = 11, 26%), community (*n* = 17, 41%), health services (*n* = 8, 19%), or a combination of both health services and university (*n* = 4, 10%), health services and community (*n* = 1, 2%) or community and university (*n* = 1, 2%) settings. All participants were referred to as females or women, with seemingly no representation of transgender, non-binary, or intersex individuals. The final included studies were published and available in English (*n* = 38, 90.5%) or Spanish only (*n* = 4, 9.5%). The studies addressed a range of MH experiences, including menstrual practices (*n* = 5, 12%), attitudes toward menstruation (*n* = 9, 21%), experiences of the menstrual cycle and bleeding characteristics (*n* = 16, 38%), PMS (*n* = 10, 24%), dysmenorrhea (*n* = 13, 31%), unclassified MH experiences (*n* = 5, 12%), and symptom management (*n* = 6, 14%). In addition, 41% of the studies (*n* = 17) explored the impact of MH experience on participation, including 12% (*n* = 5) examining the impact on academic participation, and 38% of the studies (*n* = 16) examining the impact of MH on participation in daily life.

**Table 1.** Characteristics of studies (*n* = 42).

Author(s), year of publication	Study location (remoteness)	Setting	Sample size^^^	Participant descriptor	Age range, years (mean age ± SD)	Study design (methods)	Focus of MH experience	Impact on participation
Alvarado et al. (1988)	Mexico (UK)	Community	90	Women	20–35 (22.9 ± 3.2)	Longitudinal (quantitative)	Menstrual cycle and bleeding characteristics	
Barbosa-Silva et al. (2024)	Brazil (urban and rural)	Community	**9615**	Women	18–39 (25.2 ± 6.4)*	Cross-sectional (quantitative)	Dysmenorrhea/menstrual cycle and bleeding characteristics	
Cândido et al. (2018)	Brazil (urban)	University	96	Female	18–39 (UK)	Cross-sectional (quantitative)	Dysmenorrhea/management of menstrual-related symptoms/menstrual cycle and bleeding characteristics	
Czekalski Lobascz et al. (2023)	Brazil (urban)	University	164	Women	18–41 (22.26 ± 3.21)	Cross-sectional (quantitative)	Menstrual practices/dysmenorrhea/menstrual cycle and bleeding characteristics	
de Las Mercedes Villa Rosero et al. (2022)	Ecuador (urban)	Community	2397	Women	14–49 (28.50 ± 9.04)**	Cross-sectional (quantitative)	Dysmenorrhea	
Dennerstein et al. (2010)	Brazil and Mexico (urban and rural)	Community	1048***	Women	14–50 (31.44, UK)****	Cross-sectional (quantitative)	PMS	Daily life
Diaz-Vélez et al. (2021)	Peru (rural)	Community	336	Women	15–49 (UK)	Cross-sectional (quantitative)	Dysmenorrhea/management of menstrual-related symptoms	
do Amaral et al. (2005)	Brazil (urban)	Community	64	Women	21–51 (UK)	Cross-sectional (qualitative)	Attitudes toward menstruation	
dos Santos et al. (2021)	Brazil (urban)	Health services	136	Women	19–39 (*****)	Cross-sectional (quantitative)	Dysmenorrhea/menstrual cycle and bleeding characteristics	Daily life
dos Santos et al. (2022)	Brazil (urban)	Health services	195	Women	19–49 (33.2 ± 9.1)	Cross-sectional (quantitative)	Dysmenorrhea/menstrual cycle and bleeding characteristics	
Gaybor (2019)	Argentina (urban)	Community	56	Women	20–35 (UK)	Cross-sectional (qualitative)	Menstrual practices	
Huerta-Franco and Malacara (1993)	Mexico (urban and rural)	Community	502	Female	11–51 (23.9 ± 7.1)	Cross-sectional (quantitative)	Menstrual cycle and bleeding characteristics/unclassified menstrual-related symptoms	
Ishikura et al. (2024)^# a^	Brazil (urban)	Health services^#^	96	Women	20–53 (38.1 ± 8.0)	Cross-sectional (quantitative)	Unclassified menstrual-related symptoms	Daily life
Ishikura et al. (2024)^b^	Brazil (urban)	Health services^#^	232	Women	20–53 (******)	Cross-sectional (quantitative)	Unclassified menstrual-related symptoms	Daily life
Ishikura et al. (2024)^c^	Brazil (urban)	Health services^#^	328	Women	20–53 (******)	Cross-sectional (quantitative)	Dysmenorrhea	Daily life
Khan et al. (2017)	Belize (urban and rural)	Community	267	Women	15–49 (27.9 ± 9.2)	Cross-sectional (mixed methods)*******	Menstrual practices	
Leite et al. (1994)	Brazil (urban)	University	240	Women	UK (19.9 ± UK)	Cross-sectional (quantitative)	Menstrual cycle and bleeding characteristics/unclassified menstrual-related symptoms	
Longo da Silva et al (2006)	Brazil (urban)	Community	1395	Women	15–49 (UK)	Cross-sectional	Menstrual cycle and bleeding characteristics/PMS	
Longo da Silva et al (2008)	Brazil (urban)	Community	2082	Women	22–23 (23 ± UK)	Longitudinal******** (quantitative)	Menstrual cycle and bleeding characteristics/PMS	
Makuch et al. (2011)	Brazil (urban)	Health services and University	885	Women	18–45 (UK)	Cross-sectional (quantitative)	Menstrual cycle and bleeding characteristics/attitudes towards menstruation	Academic/daily life
Makuch et al. (2012)	Brazil (urban)	Health services and university	1111	Women	18–39 (UK)	Cross-sectional (quantitative)	Menstrual cycle and bleeding characteristics/attitudes toward menstruation	
Marván and Díaz Erora (1995)	Mexico (urban and rural)	Community	100	Women	17–38 (UK)	Cross-sectional (quantitative)	PMS	
Marván et al. (1998)	Mexico (urban and rural)	Community	271	Women	17–38 (UK)	Cross-sectional (quantitative)	PMS	
Marván et al. (2005)	Mexico (urban)	University	**59**	Women	18–23 (19.48 ± UK)	Cross-sectional (quantitative)	Attitudes toward menstruation	Daily life
Marván and Lama (2009)	Mexico (urban)	University	**77**	Women	20–25 (21.18 ± UK)	Cross-sectional (quantitative)	Attitudes toward menstruation	
Marván and Trujillo (2009)	Mexico (urban and rural)	Community^+^	298	Women	19–25 (urban: 20.4, rural: 20.9, UK)	Cross-sectional (quantitative)	Attitudes toward menstruation	Daily life
Marván et al. (2014)	Mexico (urban)	Community and university	106	Women	19–46 (29.08 ± 7.97)	Cross-sectional (quantitative)	Attitudes toward menstruation	Daily life
Meza-Moreno et al. (2021)	Mexico (urban)	Community^++^	145^^^^	Women	18–33 (*********)	Case control (quantitative)	PMS	Daily life
Morales-Carmona et al. (2008)	Mexico (urban)	Health services and university	776	Women	15–35 (23.0 ± 6.0)	Cross-sectional (quantitative)	Menstrual cycle and bleeding characteristics/unclassified menstrual-related symptoms	
Ortiz et al. (2007)	Mexico (urban)	University	285	Female/women	17–33 (20.6 ± 2.4)	Cross-sectional (quantitative)	Dysmenorrhea/management of menstrual-related symptoms	
Ortiz (2010)	Mexico (urban)	University	1539	Female/women	17–35 (20.4 ± 2)	Cross-sectional (quantitative)	Dysmenorrhea/management of menstrual-related symptoms	Academic/daily life
Ortiz et al. (2022)	Mexico (urban)	University	2154	Female/women	18–33 (20.4 ± 1.9)	Cross-sectional (quantitative)	Dysmenorrhea/management of menstrual-related symptoms	Academic/Daily life
Pedro et al. (2024)	Brazil (urban and rural)	Health services	**2394**	Women	20–29 (UK)	Cross-sectional (quantitative)	PMS	
Pedrón-Nuevo et al. (1998)	Mexico (urban)	University	1066	Women	12–24 (18 ± 3.2)	Cross-sectional (quantitative)	Dysmenorrhea	Academic
Petta et al. (2010)	Brazil (urban)	Health services and University	1053	Female/women	18–40 (UK)	Cross-sectional (quantitative)	PMS/management of menstrual-related symptoms	Daily life
Rezende et al. (2022)	Brazil (urban)	University	1115	Female	Over 18 (UK; 22.5 ± 3.4)	Cross-sectional (quantitative)	PMS	Academic/daily life
Rupe et al. (2022)	Haiti (rural)	Community	200	Female	14–24 (UK)	Cross-sectional (quantitative)	Menstrual practices	
Santos et al. (2011)	Brazil (urban)	Health services	865	Women	18–45 (32 ± 8)	Cross-sectional (quantitative)	Menstrual cycle and bleeding characteristics/menstrual practices	
Snow et al. (2007)	Global—with study sites in Brazil (urban)	Health services and Community^+++^	420	Women	18–49 (UK)	Cross-sectional (quantitative)	Attitudes toward menstruation	
Szarewski et al. (2012)	Global – with study sites in Brazil (urban and rural)	Community	500	Women	15–49 (UK)	Cross-sectional (quantitative)	Attitudes toward menstruation	Daily life
Vargas-Costales et al. (2024)	Ecuador (rural and urban)	Health services	2429	Women	14–49 (24.5 ± 8.3)	Cross-sectional (quantitative)	Dysmenorrhea/menstrual cycle and bleeding characteristic	
Victor et al. (2019)	Brazil (urban)	University	649	Women	18–35 (22.1 ± 4.4)	Cross-sectional (quantitative)	Menstrual cycle and bleeding characteristics/PMS	Daily life

SD: standard deviation; UK: unknown; MH: menstrual health; PMS: premenstrual syndrome; PMDD: premenstrual dysphoric disorder.

^ In some studies, the age range exceeded the target range of 18–24 years. However, where studies provided a sample size and outcomes specific (or closer) to the 18–24 age group, only the sample size AND this age range are reported in the table and are indicated by bolded text.

^^ This study included participants with PMDD, as this condition was excluded, we only report here the sample size for those without PMDD.

* Reported mean/SD for all study participants that had a broader age range of 18 to 54.

** The reported mean & SD for the primary dysmenorrhea group is: (27.73 ± 8.63) (author-supplied data for both sets of values, including those presented in the table).

*** 548 Brazilian, 500 Mexican.

**** Mean for global sample, unknown mean and SD for Brazil and Mexico.

***** 73 with dysmenorrhea (28.4 ± 6.7), and 63 without dysmenorrhea (27.7 ± 5.9).

****** Ishikura et al. (2024b)—38.1 years (± 7.72) for the menstruating group and 35.8 years (± 8.26) for the non-menstruating group, Ishikura et al. (2024c)—non-dysmenorrhea and dysmenorrhea groups were 35.4 years (± 8.7) and 33.6 years (± 7.9). The age ranges were confirmed by the study authors for all three Ishikura (2024a,b,c studies).

******* While this study included mixed methods, the qualitative approach consisted of ‘cognitive interviews’ to qualitatively evaluate question quality, rather than to gain understanding of their MH experience, and focus groups of the interviewers, and therefore we focused solely on the quantitative data within this scoping review.

******** While this study is part of a longitudinal cohort following births in the year 1982, the findings of interest for this review only pertain to the data collected in the last follow-up in the 2004–2005 visit.

********* 67 with PMS (21.55 ± 2.32) and 78 without PMS (21.86 ± 3.05).

+ Women were recruited at their workplaces, in their homes, or in public health clinics; however, after they agreed to participate, they set up a time and place with the researcher to complete the survey; therefore, this is considered a community-based study.

++ While four women within this study were identified previously by a gynecologist in a health service to be approached for recruitment, the final setting of the study is unknown, and presumed to be community.

+++ This study was primarily conducted in health services settings, with participant recruitment occurring in gynecology and family planning outpatient clinics. In addition, community outreach was used through snowball sampling to reach a broader segment of eligible women.

# This was an epidemiologic sleep study conducted at a sleep institute (EPISONO: São Paulo Epidemiologic Sleep Study).

a How do phases of the menstrual cycle affect sleep? A polysomnographic study of the EPISONO database.

b Sleep is altered during menstruation but not inflammatory parameters: Results from polysomnography of EPISONO database.

c Altered sleep and diurnal consequences in women with dysmenorrhea: study from the EPISONO database.

The evidence-gap map in Figure 2 illustrates that most research on MH experiences among young adults in LAC regions is predominantly based on studies conducted in urban settings in Brazil, with a specific emphasis on the impacts of these experiences on daily life. Notably, of the 33 countries in the LAC region, only 21%^7^ are represented in the literature. Larger population studies tended to focus on conditions associated with the menstrual cycle, while the smaller studies focused on a range of experiences, including menstrual practices and attitudes toward menstruation.

### Synthesis of results

**Figure 3.**
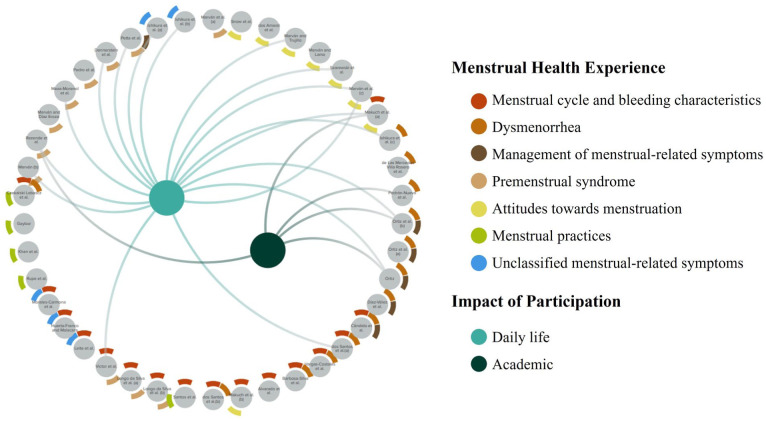
Evidence-gap map of included studies (n = 42) categorized by MH experience and impact on participation. Source: https://makrahe.kumu.io/menstrual-health-experiences-of-young-adults-in-latin-america-and-the-caribbean. MH: menstrual health.

Studies are described below according to the focus of the MH experience and the impact on participation. These are visually represented in Figure 3, which represents a static image of the interactive evidence-gap map available online.

#### Menstrual practices

Five studies examined menstrual practices.^65–69^ These studies defined menstrual practices in varying ways, focusing on the type of menstrual materials used (*n* = 3), the factors influencing material selection (*n* = 2), and one of these also included a component on access to and disposal of menstrual materials (n = 1).

Czekalski Lobascz et al.^65^ found disposable pads were the most common menstrual material among Brazilian university students (83%), a finding echoed by another Brazilian study (99.5%)^69^ and a Haitian study (92%).^67^ Although the Belize study did not specify material types, Khan et al.^68^ reported 98.3% of women had materials for period management; 97.8% of urban and 87.3% of rural women had private washing facilities. The Brazilian study identified key factors influencing material choice, rated from 1 to 10, with higher scores reflecting greater importance: product efficacy (9.56 ± 1.24), hygiene (9.42 ± 1.45), practicality (8.89 ± 1.84), vaginal health (8.80 ± 1.87), price (6.27 ± 2.92), sustainability (6.15 ± 2.98), and integrity of internal genitalia (2.76 ± 3.19).^65^ In Argentina, possible health risks associated with the use of disposable options and environmental concerns motivated participants to adopt reusable alternatives, such as menstrual cups.^66^ However, mixed feelings about menstrual cups persisted due to perceptions of genital manipulation,^65,66^ while Rupe et al.^67^ reported 40% of Haitian participants felt uncomfortable washing reusable materials in public.

#### Menstrual cycle related

Various aspects of the menstrual cycle were frequently examined across all studies. Nine studies focused on attitudes toward menstruation.^70–78^ Sixteen explored experiences of the menstrual cycle and bleeding characteristics.^65,69,71,72,79–90^ Thirteen studies included aspects of dysmenorrhea,^24,27,65,81–84,89,91–95^ 10 investigated PMS,^54,87,88,90,96–101^ and 5 reported MH experiences that were not otherwise classified.^80,85,86,102,103^ All but one of these studies utilized quantitative designs.

Menstrual bleeding was predominantly viewed negatively in Brazilian studies.^70–72,76,78^ Between 61% and 64.3% of women disliked menstruation, citing inconvenience and discomfort.^71,72^ Around 60% wished to postpone menstruation,^76^ and one-third preferred ‘never bleeding’.^78^ Qualitative findings echoed these sentiments, with some describing menstruation as an ‘unnecessary nuisance’ and supporting suppression.^70^ However, a minority valued menstruation as a sign of health (37.4%) or as an indicator of non-pregnancy (33.9%),^71^ and others linked it to femininity, youth, and fertility.^70^

In Mexico, similar attitudes toward menstrual suppression were noted, with only 20.8% of undergraduate students preferring to menstruate monthly.^75^ Mean ‘annoyance’ scores on the BATM ranged from 3.65 to 3.69 among urban women and 3.78 among rural women (rated on a five-point Likert scale, from 1 – disagree strongly to 5 – agree strongly), indicating moderate annoyance with menstruation in young women.^73,74^ Women with higher annoyance scores also reported more negative feelings toward menstruation (measured by the MDQ subscale) (*p* < 0.01).^77^ For the BATM’s ‘secrecy’ factor, urban women’s mean scores ranged from 2.08 to 2.33, while rural women scored 2.69, suggesting rural women were more likely to believe menstruation should remain private (*p* < 0.001).^73,74^ The theme of secrecy, while prominent in these findings, was not widely addressed in other studies.

General menstrual cycle and bleeding characteristics were included across 16 studies. Some studies excluded these details due to inclusion criteria, such as having a regular menstrual cycle to be considered a participant. Among studies that reported data on regular menstrual cycle lengths (defined as 21–35 days), the prevalence of regular cycles varied widely, from 26% to 93.6%.^65,79,81,82,84,86–90^

Reported bleeding duration also varied across studies. In one study, 99% of participants experienced bleeding between 1 and 6 days,^79^ other studies reported 3–7 days for 78.8%,^80^ 3–5 days for 79.9%,^85^ 4–5 days for 58.3%^71^ and 58.4% of participants.^72^ One study simply noted over 3 days for 78% ^65^ and another study under 7 days for 71.8% of participants.^82^ Average menstrual duration was reported at 4.4 days,^83^ and 4.9 ± 1.9 among those with dysmenorrhea.^89^

Among the reviewed dysmenorrhea studies, prevalence varied widely, ranging from 6% to 98.8%.^65,82^ In Brazil, Cândido et al.^82^ reported 6% prevalence among female university students, while Czekalski Lobascz et al.^65^ observed a significantly higher prevalence of 98.8%. Other Brazilian studies indicated moderate prevalence, including 72% with absent, mild, or moderate symptoms (30% absent or mild, 42% moderate).^81^ 56%^84^ and 54% were reported among women health service users.^83^ In Ecuador, prevalence ranged from 8.9%^91^ to 26.6%,^89^ while in Peru, 19% of women reported moderate pain.^95^ In Mexico, prevalence varied across university samples, including 52.1%, 62.4%, 64%, and 78.9%.^24,27,93,94^

Half of the PMS studies reviewed reported prevalence among Brazilian populations, with findings varying significantly. Among female university students, PMS prevalence was reported at 46.7%^96^ and 49.9% in a separate study.^90^ In a combined health and university setting, Petta et al.^54^ found a prevalence of 60.3%. Community-based studies showed similar variation: 60.3% of women self-reported PMS, with 25.2% meeting criteria based on modified Diagnostic and Statistical Manual of Mental Disorders, Fourth Edition scores requiring five or more symptoms.^87^ Another study reported 62.2% self-reported PMS, while 13.4% experienced ‘moderate PMS’, defined as at least eight symptoms causing significant relationship impairment.^88^

Two Mexican community studies assessed PMS in rural and urban areas using the MDQ, focusing on symptom severity rather than prevalence. In the first study, 84% of rural women reported mild psychological-behavioral symptoms, with 8% reporting severe symptoms, compared to 64% mild and 32% severe in urban women.^101^ Physical symptoms were mild in 64% of rural and 70% of urban participants, with severe symptoms reported by 30% in both groups.^101^ The second study found urban women, particularly those engaged in professional studies, experienced more severe premenstrual changes across multiple symptom areas compared to rural women, where a higher proportion reported no symptoms.^100^

In a study of 20–29 year old Brazilian women, participants were eligible only if they reported functional impairment linked to PMS symptoms.^98^ Highly prevalent symptoms included anxiety or tension (98.4%), irritability or anger (98.2%), and depression or sadness (94.0%).

A community study in Mexico found that 35.8% reported at least one physical or emotional symptom during their menstrual cycle, which intensified in the late luteal phase, though these were not explicitly classified as PMS or dysmenorrhea.^85^ Among participants with eumenorrhea, 19.5% scored as ‘cases’ on the General Health Questionnaire, indicating psychological distress despite typical menstrual patterns.^80^ A Brazilian university study noted that most young women experienced physical discomfort during menstruation, with 40% reporting cramps and common premenstrual mood changes such as irritability and anxiety, although dysmenorrhea and PMS were not specifically mentioned.^86^ Another Brazilian study found mild anxiety across menstrual cycle phases, with Beck Anxiety Inventory scores of 9.2 (SD ± 8.7) in the follicular phase, 9.1 (SD ± 7.4) in the luteal phase, and 9.7 (SD ± 8.0) during menstruation.^102^ These scores were similar in non-menstruating (9.69, SD ± 8.69) and menstruating groups (10.9, SD ± 9.81).^103^

#### Management of menstrual-related symptoms

Six studies reported strategies for managing menstrual-related symptoms.^24,27,54,82,93,95^ Brazilian and Mexican studies highlighted various coping strategies for menstrual disorders. In Mexico, three studies found that only 25.9% to 33.5% of women with dysmenorrhea sought medical consultation.^24,27,93^ Although 78.7% used prescribed medications to alleviate pain, the overall effectiveness was limited, with only 18.4% reporting complete relief from symptoms.^24^

Self-medication was common, with 61.7% to 64.9% of female students engaging in this practice, averaging 6.0 ± 3.6 to 6.1 ± 3.8 cycles per year.^24,93^ Paracetamol was frequently used and demonstrated statistically significant effectiveness compared to other options (*p* = 0.017).^24^ Nonsteroidal anti-inflammatory drugs were also commonly used by those with dysmenorrhea.^82^ In urban Brazil, medication was the primary approach for managing PMS (33.3%), supplemented by relaxation and distraction techniques.^54^ In rural Peru, 82.1% of women with dysmenorrhea reported using alternative and complementary treatments, followed by medication use (11.6%).^95^

#### Impact on participation

##### Academic

Five studies from Brazil and Mexico examined the academic impacts of menstruation, including absenteeism (*n* = 3)^24,27,94^ and general interference with academic activities (*n* = 5).^24,27,71,94,96^ Dennerstein et al.^97^ and Rupe et al.^67^ also reported impacts but included a broader age range (14–50 years and 14–24 years, respectively), making it unclear if the findings pertain to university or secondary school students, and are thus not included here. Among Mexican students with dysmenorrhea, 37.0% reported absenteeism across 2.6 ± 2.1 menstrual cycles per year,^27^ while another study noted 42.1% absenteeism in 3.1 ± 2.2 cycles.^24^ A separate Mexican study found 19.8% absenteeism in 20–24 year olds.^94^

In Brazil, 22.2% of students reported menstrual interference with academic tasks.^71^ Over 30% of Brazilian students with PMS reported that their symptoms moderately to severely hindered their academic productivity.^96^ In addition, 78.7% of students with dysmenorrhea experienced concentration difficulties, 69.0% reported a decline in performance, and 49.3% believed their grades would improve without dysmenorrhea.^27^

##### Daily life

Sixteen quantitative studies from Brazil and Mexico included a component examining the impact of MH experiences on daily life.^24,27,54,71,73,74,76,77,83,90,92,96,97,99,102,103^ Despite variations in measurement instruments and outcome variables, these studies are grouped below according to their focus on MH experiences for clarity and consistency.

In studies on attitudes toward menstruation, 27% of Brazilian women agreed that menstruation has a severe negative impact on their daily lives.^76^ In a separate study, 43.3% of Brazilian women indicated that menstruation adversely affected their relationships with partners, although the impact was reported to be less pronounced on family relationships, social, and professional interactions.^71^ Among Mexican young women, there was a somewhat neutral stance or slight disagreement regarding menstruation affecting daily activities, with a mean score of 2.23 (SD = 0.86) on the disability factor of the BATM.^73^ A separate Mexican study showed similar findings between urban and rural women, with mean scores of 2.4 ± 0.99 and 2.28 ± 1.25, respectively.^74^ In another Mexican study, higher agreement with the idea that menstruation should be kept secret was associated with increased reports of behavioral change (MDQ subscale) (*p* < 0.01), indicating modifications in activities or social interactions due to discomfort or avoidance related to menstruation.^77^

Among Mexican university students, 65% to 90.4% reported that dysmenorrhea limited their daily life, affecting 4.2 ± 2.9 to 4.4 ± 3.3 cycles per year.^24,27^ In Brazil, women with severe pain had greater difficulties with mobility (*p* = 0.003) and community participation (*p* = 0.030) compared to those with mild-to-moderate pain.^83^ However, functional capacities did not significantly differ between those with and without dysmenorrhea, based on the World Health Disability Assessment Schedule.^83^ A separate Brazilian study using the World Health Organization Quality of Life (WHOQOL) BREF questionnaire reported lower physical domain scores in women with dysmenorrhea (62.5 ± 16.3) compared to those without dysmenorrhea (69.4 ± 16.6, *p* = 0.047, and Cohen’s *d* = 0.4).^92^ Another Brazilian study reported physical domain scores for healthy, non-dysmenorrheic women across menstrual phases as follows: 60.4 (SD ± 11.2) in the follicular phase, 60.8 (SD ± 10.0) in the luteal phase, and 57.6 (SD ± 10.6) in the menstrual phase.^102^ For women during the menstrual phase versus those in another phase, the scores were 52 (SD ± 10.48) and 57.6 (SD ± 10.76), respectively.^103^ Among Brazilian students with unclassified menstrual symptoms, 8% reported canceling engagements or limiting their activities to only necessary tasks during times of physical discomfort.^86^

Among PMS studies, the community study conducted by Dennerstein et al.^97^ revealed that 21.5% of Brazilian and 14.4% of Mexican participants with PMS reported that menstrual symptoms interfered with their daily life more generally. In another Brazilian study, women with PMS reported their symptoms affected partner relationships (58.9%) and family interactions (52.8%), as well as domestic, professional, and social activities (42.3%).^54^ Moderate to severe impacts on family relationships and professional and social activities were also highlighted by Rezende et al.^96^ Furthermore, Victor et al.^90^ identified through the WHOQOL BREF questionnaire significant differences in mobility and community participation domains between students experiencing mild PMS and those without PMS (*p* = 0.010 and *p* = 0.009, respectively). In Mexico, women with PMS had a significantly higher PSST total score (20.13 ± 7.10) compared to the group without diagnosis (15.86 ± 8.51) (*p* < 0.001).^99^ Specifically, daily-life impairment was significantly greater in the PMS group (6.46 ± 2.29) compared to the group without diagnosis (2.56 ± 1.98) (*p* < 0.001).^99^

## Discussion

This scoping review summarized evidence from 42 studies, with the majority coming from Brazil (50%) and Mexico (33%). This pattern likely reflects the presence of more research infrastructure and funding in these countries, as evidenced by the concentration of studies documenting diverse research efforts. Brazil’s comparatively high investment in research and development, reportedly quadrupling that of Mexico, may also contribute to its prominence in the literature.^104^ As a result, the findings are geographically skewed, and thematic saturation cannot be assumed.

Moreover, key experiences in underrepresented countries or marginalized communities may differ significantly from what is currently documented, with 69% of studies set in urban locations. This concentration leaves large portions of the region, especially rural areas, understudied. While rural MH has increasingly gained attention globally, as seen in Kashyap and Choudhari’s^105^ review of 40 rural-focused studies, namely in India and Rossouw and Ross’^106^ analysis of rural–urban disparities across eight low- and middle-income countries, it seems the LAC region has not mirrored this trend. Nonetheless, we see similar broader infrastructural and sociocultural trends, for example, in our review, rural women reported approximately 10% lower access to private washing facilities,^68^ a higher tendency to keep menstruation secret,^74^ and greater use of complementary medicines.^95^ These findings underscore the importance of context-specific research, as the true extent and impact of MH within these populations remain unclear. Addressing this gap is essential for developing inclusive and contextually relevant MH policies in LAC.

While global reviews have similarly found that menstruation adversely affects academic and daily life,^13,14,26^ only five of the 42 included studies in this review addressed the impact on academic participation with varying instruments for data collection. For example, one study used a visual analog scale adapted from pain assessment tools,^71^ another applied the PSST,^96^ and a third used a custom questionnaire to explore concentration and performance impacts.^27^ These differences may reflect a broader challenge in MH research: the lack of standardized instruments for assessing academic outcomes. Munro et al.^13^ global review similarly noted that while many studies reported impacts on absenteeism, concentration, and academic performance, how these outcomes were measured varied. Tools ranged from general self-report items to menstrual diaries and symptom interference scales, with no studies validating student absentee responses against official academic records. The diversity of university environments, spanning disciplines, teaching formats, and attendance expectations further complicates efforts to systematically evaluate the academic impact of menstruation. Together, these factors could contribute to both the underrepresentation of higher education contexts in MH research in LAC and the limited understanding of how MH influences academic participation.

Notably, few studies employed longitudinal or qualitative methods, limiting insight into how MH experiences evolve over time or intersect with life transitions such as entering university or employment. While cross-sectional and quantitative studies can identify prevalence and associations at a single point in time, they often miss the dynamic, cumulative, and contextual nature of MH experiences. By contrast, longitudinal research from high-income countries has shown how symptoms like menstrual pain and heavy bleeding can intensify over time and impact broader aspects of well-being, informing early screening and long-term care strategies.^107,108^ Qualitative studies offer depth and nuance, revealing how stigma, emotional discomfort, and social norms shape daily life and delay care, insights that have led to calls for integrated screening and stigma-reduction in primary healthcare.^109^ Similar research is urgently needed in LAC to generate context-specific clinical and public health recommendations, alongside inclusive MH interventions that reflect the lived realities.

Product use patterns, particularly the widespread preference for disposable pads, align with findings among Latinas in the United States.^110^ However, only two LAC studies explored the reasons behind menstrual product or material choices.^65,66^ Broader literature suggests these decisions are shaped by cost, cultural norms, access, and environmental concerns.^26^ Importantly, several LAC countries, including Mexico, Ecuador, and Barbados, have recently eliminated menstrual product taxes.^111^ Yet, over half still impose such discriminatory taxation, with an average rate of 5.8% across the region.^111^ However, although tax removal is often framed as a step toward menstrual equity, its impact may be overstated. As King^112^ notes, similar campaigns in the United Kingdom have unintentionally boosted profits for disposable product manufacturers without meaningfully reducing costs for consumers. These efforts rarely challenge the underlying market monopolies or profit structures that keep prices high, and they often overlook more sustainable, cost-effective alternatives like menstrual cups. Despite this, none of the included LAC studies explicitly examined how product affordability or access influences participation in higher education or work, an important gap in understanding the full impact of period poverty (defined as inadequate access to menstrual products and basic WASH infrastructure)^113^ on educational and economic inclusion.

Participation in higher education may also be influenced by menstrual stigma, particularly around secrecy and concealment.^11,114^ Gender ideologies like machismo and marianismo in Latin America, which emphasize male dominance and female purity, could reinforce the notion of menstruation as shameful or impure.^115^ While few studies explicitly link such beliefs to academic or daily life impacts,^116^ our review suggests a generational shift. Younger urban women generally rejected the idea that menstruation should be kept secret.^73,74^ The same Marván et al.^73^ study indicated that younger men also disagreed, whereas middle-aged participants tended to agree. These evolving attitudes signal potential for challenging and reducing entrenched stigma. Evidence from other contexts suggests that multifaceted approaches, including education-based interventions, alongside community engagement, may be more effective in reducing menstrual stigma by increasing bodily autonomy through MH literacy, fostering supportive environments, and challenging gendered power dynamics.^117,118^

Furthermore, a critical omission is the complete lack of studies on gender-diverse individuals. This gap is not unique to LAC, and global MH research often overlooks gender-diverse populations.^13^ However, this is particularly troubling in the LAC region, where rising political tensions, anti-gender rhetoric, and widespread violence continue to marginalize sexual and gender minority communities. Unprosecuted hate crimes and experiences of systemic discrimination in healthcare, education, and daily life remain prevalent for lesbian, gay, bisexual, transgender, and queer students across Latin America.^119^ Inclusive research that addresses the specific MH needs of all young people, regardless of gender identity, is essential to informing equitable policies.

Finally, healthcare access for menstruation-related concerns remains limited. Across the reviewed studies, while many participants reported symptoms like dysmenorrhea, few sought care,^24,27^ and even fewer found relief.^24^ While health systems across the LAC region vary significantly and face major challenges, including access to sexual and reproductive health, limited health insurance schemes, and a high financial burden of accessing care,^30^ these limitations are not unique to the region. European research, for instance, has documented issues such as feeling unheard by providers and dissatisfaction with treatment,^120^ which are issues reported across LAC’s fragmented health systems. Identifying these barriers is essential for developing targeted interventions to mitigate their impact, highlighting the need for further research, while also continuing to address the broader implications of healthcare access to support gender equality across LAC.

In sum, this review reveals that while MH research in LAC has grown, it remains geographically and thematically narrow. To strengthen the evidence base, future research must:

1. Expand into rural areas and underrepresented countries,
2. Develop and apply standardized, validated tools to measure MH outcomes in higher education settings,
3. Incorporate longitudinal study designs and qualitative methods,
4. Explore menstrual material/product access (including its influence on higher education or work),
5. Include transgender, gender-diverse, and intersex people, and
6. Explore healthcare experiences for MH.

Our findings also underscore the need to implement stigma-reduction campaigns through education and community engagement, and improve access to culturally sensitive, gender-inclusive healthcare. Advancing these priorities will help ensure that MH initiatives, interventions and policies reflect the diverse needs of young people PFAB across LAC and promote greater equity and inclusion.

### Strengths and limitations

Despite a comprehensive search strategy, some relevant studies may have been missed due to inconsistent terminology and classification of MH conditions. This review included studies on dysmenorrhea and PMS, including those that did not specify whether dysmenorrhea was primary or secondary, or whether PMS severity was mild or moderate. While PD and mild-to-moderate PMS were within scope, studies explicitly focused on secondary dysmenorrhea, often associated with underlying conditions like endometriosis,^121^ and PMDD, a severe form of PMS affecting 3%–8% of the population,^122^ were excluded. This inclusive approach aimed to capture a broader range of MH experiences by accommodating studies that may not use clinical classifications, while still maintaining a focus on common, non-pathological symptoms.

We also included relevant components from studies, even when some outcomes did not meet our inclusion criteria. For example, studies focusing on CPP were excluded due to the lower prevalence of this condition (< 26.6%).^49^ However, since dysmenorrhea is a common cause of cyclic CPP, we included two Ecuadorian studies^89,91^ that identified PD as a subtype of CPP, and we analyzed only the PD-related findings. Similarly, in the study by Meza-Moreno et al.,^99^ which addressed healthy menstruating individuals, PMS, and PMDD, we focused solely on the data related to healthy menstruating individuals and PMS. We took a similar approach for studies that reported on eumenorrhea and menorrhagia, and one survey validation study that also included a separate qualitative component. This approach allowed us to capture a wider range of MH experiences while maintaining focus on our primary outcomes.

Our review primarily examined MH experiences across a broad range of young adult populations. However, we acknowledge the importance of studies that focus on specific indigenous groups, such as Aymara women in Northern Chile,^123^ whose experiences are influenced by unique sociocultural and generational factors. While we included studies with participants from broader cohorts, many of whom are Mestizo, reflecting the region’s mixed ancestry, this approach may limit the representation of specific Indigenous perspectives. As such, we recommend that future research place greater emphasis on distinct Indigenous perspectives to further enrich our understanding within the region.

Finally, our scope excluded studies conducted in secondary school settings to maintain our focus on young adults. In cases where the educational setting was ambiguous, we used contextual information to make inferences. For example, in the study by Pedrón-Nuevo et al.,^94^ although the setting was not explicitly stated, participants aged 20–24 were assumed to be university students. Similarly, Morales-Carmona et al.^80^ noted recruitment from various settings, including secondary schools, but the mean age of 23 suggested that most participants were not secondary school attendees.

## Conclusion

This scoping review provides a critical overview of MH experiences among young adults in the LAC region, highlighting both the growth and limitations of current research. Understanding these experiences is essential for addressing gender inequalities in health, education, and economic participation. The review identifies significant gaps in geographic coverage, methodological diversity, and population inclusion, particularly the underrepresentation of rural communities, transgender and nonbinary individuals, and the impact on participation in academia for university students. Addressing these gaps is vital to inform inclusive, context-specific initiatives, interventions and policies that promote MH equity and support the well-being and opportunities of young people PFAB across LAC.

## Supplemental Material

sj-docx-1-whe-10.1177_17455057251379612 – Supplemental material for Menstrual health among young adults in Latin America and the Caribbean: A scoping review and evidence-gap map Supplemental material, sj-docx-1-whe-10.1177_17455057251379612 for Menstrual health among young adults in Latin America and the Caribbean: A scoping review and evidence-gap map by Lisa Irene Jones, Michelle A. Krahe, Nicola Rahman, Neil Harris, Nicola Wiseman and Gabriela Bustamante in Women's Health

sj-docx-2-whe-10.1177_17455057251379612 – Supplemental material for Menstrual health among young adults in Latin America and the Caribbean: A scoping review and evidence-gap map Supplemental material, sj-docx-2-whe-10.1177_17455057251379612 for Menstrual health among young adults in Latin America and the Caribbean: A scoping review and evidence-gap map by Lisa Irene Jones, Michelle A. Krahe, Nicola Rahman, Neil Harris, Nicola Wiseman and Gabriela Bustamante in Women's Health

sj-docx-3-whe-10.1177_17455057251379612 – Supplemental material for Menstrual health among young adults in Latin America and the Caribbean: A scoping review and evidence-gap map Supplemental material, sj-docx-3-whe-10.1177_17455057251379612 for Menstrual health among young adults in Latin America and the Caribbean: A scoping review and evidence-gap map by Lisa Irene Jones, Michelle A. Krahe, Nicola Rahman, Neil Harris, Nicola Wiseman and Gabriela Bustamante in Women's Health

## References

[1] Schoep ME Nieboer TE van der Zanden M , et al. The impact of menstrual symptoms on everyday life: a survey among 42,879 women. Am J Obstet Gynecol 2019; 220(6): 569.e1–569.e7. 10.1016/j.ajog.2019.02.048 30885768

[2] The World Bank. Menstrual health and hygiene, https://www.worldbank.org/en/topic/water/brief/menstrual-health-and-hygiene (2022).

[3] Hennegan J Winkler IT Bobel C , et al. Menstrual health: a definition for policy, practice, and research. Sex Reprod Health Matters 2021; 29(1): 1911618. 33910492 10.1080/26410397.2021.1911618 PMC8098749

[4] Elledge MF Muralidharan A Parker A , et al. Menstrual hygiene management and waste disposal in low and middle income countries—a review of the literature. Int J Environ Res Public Health 2018; 15(11): 2562. 30445767 10.3390/ijerph15112562 PMC6266558

[5] Sumpter C Torondel B. A systematic review of the health and social effects of menstrual hygiene management. PLoS One 2013; 8(4): e62004. 10.1371/journal.pone.0062004 PMC3637379 23637945

[6] Hennegan J Montgomery P. Do menstrual hygiene management interventions improve education and psychosocial outcomes for women and girls in low and middle income countries? A systematic review. PLoS One 2016; 11(2): e0146985. 10.1371/journal.pone.0146985 PMC4749306 26862750

[7] Coast E Lattof SR Strong J. Puberty and menstruation knowledge among young adolescents in low- and middle-income countries: a scoping review. Int J Public Health 2019; 64(2): 293–304. 30740629 10.1007/s00038-019-01209-0 PMC6439145

[8] Shannon AK Melendez-Torres GJ Hennegan J. How do women and girls experience menstrual health interventions in low- and middle-income countries? Insights from a systematic review and qualitative metasynthesis. Culture Health Sex 2021; 23(5): 624–643. 10.1080/13691058.2020.1718758 32116149

[9] Rastogi S Khanna A Mathur P. Educational interventions to improve menstrual health: approaches and challenges. Int J Adolesc Med Health 2019; 33: 0024. 10.1515/ijamh-2019-0024 31136299

[10] Kuhlmann AS Henry K Wall LL. Menstrual hygiene management in resource-poor countries. Obstet Gynecol Survey 2017; 72(6): 356–376. 10.1097/OGX.0000000000000443 PMC5482567 28661550

[11] Sommer M Chandraratna S Cavill S , et al. Managing menstruation in the workplace: an overlooked issue in low- and middle-income countries. Int J Equity Health 2016; 15: 86. 27268416 10.1186/s12939-016-0379-8 PMC4895811

[12] Rod NH Davies M de Vries TR , et al. Young adulthood: a transitional period with lifelong implications for health and wellbeing. BMC Global Public Health 2025; 3(1): 25. 40140902 10.1186/s44263-025-00148-8 PMC11948773

[13] Munro AK Hunter EC Hossain SZ , et al. A systematic review of the menstrual experiences of university students and the impacts on their education: a global perspective. PLoS One 2021; 16(9): e0257333. 10.1371/journal.pone.0257333 PMC8432759 34506544

[14] Armour M Parry K Manohar N , et al. The prevalence and academic impact of dysmenorrhea in 21,573 young women: a systematic review and meta-analysis. J Womens Health 2019; 28(8): 1161–1171. 10.1089/jwh.2018.7615 31170024

[15] Wang J Conwell J. Higher education and health at midlife: evaluating the role of college quality. SSM—Population Health 2022; 19: 101228. 36164493 10.1016/j.ssmph.2022.101228 PMC9508472

[16] Powdthavee N Lekfuangfu WN Wooden M. What’s the good of education on our overall quality of life? A simultaneous equation model of education and life satisfaction for Australia. J Behav Exper Econ 2015; 54: 10–21. 28713668 10.1016/j.socec.2014.11.002 PMC5510659

[17] Zajacova A Lawrence EM. The relationship between education and health: reducing disparities through a contextual approach. Ann Rev Public Health 2018; 39: 273–289. 29328865 10.1146/annurev-publhealth-031816-044628 PMC5880718

[18] Paddock-Hall K. Navigating healthcare in higher education for students with gynaecological pain: an exploratory survey. BMC Womens Health 2025; 25(1): 334. 40618049 10.1186/s12905-025-03882-1 PMC12229034

[19] MacGregor B Allaire C Bedaiwy MA , et al. Disease burden of dysmenorrhea: impact on life course potential. Int J Womens Health 2023; 15: 499–509. 37033122 10.2147/IJWH.S380006 PMC10081671

[20] Nabilal Patavegar B Ahmad S Hassan SB , et al. A study of school absenteeism during menstruation amongst adolescent school girls in an urban area of North India. Nat J Commun Med 2024; 15(9): 741–746.

[21] Femi-Agboola DM Sekoni OO Goodman OO. Dysmenorrhea and its effects on school absenteeism and school activities among adolescents in selected secondary schools in Ibadan, Nigeria. Niger Med J 2017; 58: 143–148. 31057207 10.4103/nmj.NMJ_47_17 PMC6496977

[22] Orhan C Çelenay ŞT Demirtürk F , et al. Effects of menstrual pain on the academic performance and participation in sports and social activities in Turkish university students with primary dysmenorrhea: a case control study. J Obstet Gynaecol Res 2018; 44(11): 2101–2109. 30043399 10.1111/jog.13768

[23] Bajalan Z Moafi F MoradiBaglooei M , et al. Mental health and primary dysmenorrhea: a systematic review. J Psychosom Obstet Gynaecol 2019; 40(3): 185–194. 29745745 10.1080/0167482X.2018.1470619

[24] Ortiz MI. Primary dysmenorrhea among Mexican university students: prevalence, impact and treatment. Euro J Obstet Gynecol Reprod Biol 2010; 152(1): 73–77. 10.1016/j.ejogrb.2010.04.015 20478651

[25] Ortiz MI Rangel-Flores E Carrillo-Alarcón LC , et al. Prevalence and impact of primary dysmenorrhea among Mexican high school students. Int J Gynecol Obstet 2009; 107(3): 240–243. 10.1016/j.ijgo.2009.07.031 19716130

[26] Hennegan J Shannon AK Rubli J , et al. Women’s and girls’ experiences of menstruation in low- and middle-income countries: a systematic review and qualitative metasynthesis. PLoS Medicine 2019; 16(5): e1002803. 10.1371/journal.pmed.1002803 PMC6521998 31095568

[27] Ortiz MI Espinoza-Ramírez AL Cariño-Cortés R , et al. Impact of primary dysmenorrhea on the academic performance of university students. Enferm Clín (Engl Ed) 2022; 32(5): 351–357. 36085001 10.1016/j.enfcle.2021.12.007

[28] The World Bank. The World Bank in Latin America and the Caribbean. World Bank Group, https://www.worldbank.org/en/region/lac (2023).

[29] Oliveira VC Pena ÉD Andrade GN , et al. Menstrual hygiene access and practices in Latin America: scoping review. Revista Latino-Am Enfermagem 2023; 31: e4028. 10.1590/1518-8345.6736.4029 PMC10594935 37878965

[30] Ruano AL Rodríguez D Rossi PG , et al. Understanding inequities in health and health systems in Latin America and the Caribbean: a thematic series. Int J Equity Health 2021; 20(1): 94. 33823879 10.1186/s12939-021-01426-1 PMC8023548

[31] Gilardino RE Valanzasca P Rifkin SB. Has Latin America achieved universal health coverage yet? Lessons from four countries. Arch Public Health 2022; 80(1): 38. 35063033 10.1186/s13690-022-00793-7 PMC8777418

[32] Frenk J Gómez-Dantés O. Health systems in Latin America: the search for universal health coverage. Arch Med Res 2018; 49(2): 79–83. 29960828 10.1016/j.arcmed.2018.06.002

[33] Arksey H O’Malley L. Scoping studies: towards a methodological framework. Int J Soc Res Methodol 2005; 8(1): 19–32.

[34] Tricco AC Lillie E Zarin W , et al. PRISMA extension for scoping reviews (PRISMA-ScR): checklist and explanation. Ann Int Med 2018; 169(7): 467–473. 30178033 10.7326/M18-0850

[35] Aromataris E Munn Z. 11.2.2 Developing the title and question. JBI, 2020.

[36] Brooks-Gunn J Ruble DN. The menstrual attitude questionnaire. Psychosom Med 1980; 42(5): 503–512. 7465737 10.1097/00006842-198009000-00005

[37] Moos RH. The development of a Menstrual Distress Questionnaire. Psychosom Med 1968; 30(6): 853–867. 5749738 10.1097/00006842-196811000-00006

[38] Marván ML Ramírez-Esparza D Cortés-Iniestra S , et al. Development of a new scale to measure Beliefs about and Attitudes Toward Menstruation (BATM): data from Mexico and the United States. Health Care Women Int 2006; 27(5): 453–473. 16877294 10.1080/07399330600629658

[39] Steiner M Macdougall M Brown E. The Premenstrual Symptoms Screening Tool (PSST) for clinicians. Arch Womens Mental Health 2003; 6(3): 203–209. 10.1007/s00737-003-0018-4 12920618

[40] Carney CE Edinger JD Meyer B , et al. Daily activities and sleep quality in college students. Chronobiol Int 2006; 23(3): 623–637. 16753946 10.1080/07420520600650695

[41] Amornsriwatanakul A Rahman HA Wattanapisit A , et al. University students’ overall and domain-specific physical activity during COVID-19: a cross-sectional study in seven ASEAN countries. Heliyon 2022; 8(12): e12466. 10.1016/j.heliyon.2022.e12466 PMC9760595 36568666

[42] Alarcon R Ordoñez-Mancheno J Velásquez E , et al. A scoping review of the Venezuelan migration in three South American countries: sociocultural and mental health perspectives. World Social Psychiatry 2022; 4(1): 13–23.

[43] Shamsuddin M Acosta PA Battaglin Schwengber R , et al. Integration of Venezuelan refugees and migrants in Brazil, The World Bank, 2021, p.63.

[44] United Nations Children’s Fund. Guidance note: menstrual health and hygiene for girls and women with disabilities, https://www.unicef.org/media/98881/file/MHH-Disabilities-Guidance-Note-ENG.pdf

[45] Zhang S Gong TT Wang HY , et al. Global, regional, and national endometriosis trends from 1990 to 2017. Ann N Y Acad Sci 2021; 1484(1): 90–101. 32909625 10.1111/nyas.14468

[46] Deswal R Narwal V Dang A , et al. The prevalence of polycystic ovary syndrome: a brief systematic review. J Human Reprod Sci 2020; 13(4): 261–271. 33627974 10.4103/jhrs.JHRS_95_18 PMC7879843

[47] El-Hemaidi I Gharaibeh A Shehata H. Menorrhagia and bleeding disorders. Curr Opin Obstet Gynecol 2007; 19(6): 513–520. 18007127 10.1097/GCO.0b013e3282f1ddbe

[48] Kitahara Y Hiraike O Ishikawa H , et al. National survey of abnormal uterine bleeding according to the FIGO classification in Japan. J Obstet Gynaecol Res 2023; 49(1): 321–330. 36258286 10.1111/jog.15464

[49] Ahangari A. Prevalence of chronic pelvic pain among women: an updated review. Pain Phys 2014; 17(2): E141–E147. 24658485

[50] Harlow SD Windham GC Paramsothy P. Menstruation and menstrual disorders: the epidemiology of menstruation and menstrual dysfunction. In: Goldman MB Troisi R Rexrode KM (eds) Women and health. 2nd ed. Academic Press, 2013, pp.163–177.

[51] Schiola A Lowin J Lindemann M , et al. The burden of moderate/severe premenstrual syndrome and premenstrual dysphoric disorder in a cohort of Latin American Women. Value Health 2011; 14(5): S93–S95. 10.1016/j.jval.2011.05.008 21839909

[52] Walker MH Coffey W Borger J. Menorrhagia. StatPearls, 2024. 30725595

[53] Whitaker L Critchley HOD . Abnormal uterine bleeding. Best Pract Res Clin Obstet Gynaecol 2016; 34: 54–65. 26803558 10.1016/j.bpobgyn.2015.11.012 PMC4970656

[54] Petta CA Osis MJD de Pádua KS , et al. Premenstrual syndrome as reported by Brazilian women. Int J Gynaecol Obstet 2010; 108(1): 40–43. 19892346 10.1016/j.ijgo.2009.07.041

[55] Bakhsh H Algenaimi E Aldhuwayhi R , et al. Prevalence of dysmenorrhea among reproductive age group in Saudi Women. BMC Womens Health 2022; 22(1): 78. 35305636 10.1186/s12905-022-01654-9 PMC8933932

[56] Kural M Noor NN Pandit D , et al. Menstrual characteristics and prevalence of dysmenorrhea in college going girls. J Family Med Primary Care 2015; 4(3): 426–431. 26288786 10.4103/2249-4863.161345 PMC4535108

[57] Abu Alwafa R Badrasawi M Haj Hamad R . Prevalence of premenstrual syndrome and its association with psychosocial and lifestyle variables: a cross-sectional study from Palestine. BMC Womens Health 2021; 21(1): 233. 34090416 10.1186/s12905-021-01374-6 PMC8178841

[58] Conzatti M Perez AV Maciel RF , et al. Sleep quality and excessive daytime sleepiness in women with premenstrual syndrome. Gynecol Endocrinol 2021; 37(10): 945–949. 34409910 10.1080/09513590.2021.1968820

[59] Conzatti M Maciel RF Perez AV , et al. Premenstrual syndrome and female sexual function. J Sex Marital Therap 2021; 47(2): 186–196. 33302813 10.1080/0092623X.2020.1856988

[60] Bramer WM Giustini D de Jonge GB , et al. De-duplication of database search results for systematic reviews in EndNote. J Med Library Assoc 2016; 104(3): 240–243. 10.3163/1536-5050.104.3.014 PMC4915647 27366130

[61] Gore G. Deduplicating in EndNote, https://libraryguides.mcgill.ca/epib629/deduplicating#s-lg-box-16286927 (2023).

[62] Veritas Health Innovation. Covidence systematic review software. Veritas Health Innovation, www.covidence.org (2025).

[63] Kumu. Relationship mapping software. Kumu Inc, 2025.

[64] Santos-Silva R Tufik S Conway SG , et al. Sao Paulo Epidemiologic Sleep Study: rationale, design, sampling, and procedures. Sleep Med 2009; 10(6): 679–685. 19230759 10.1016/j.sleep.2008.11.001

[65] Czekalski Lobascz B de França Reis MB de Perez Monteiro e Tiburcio Mendes G , et al. Determinants of menstrual cup use among undergraduate medical students: a cross-sectional study. Int J Gynecol Obstet 2023; 160(3): 1007–1011. 10.1002/ijgo.14450 36087018

[66] Gaybor J. Empowerment, destigmatization and sustainability: the co-construction of reusable menstrual technologies in the context of menstrual activism in Argentina. Gender Tech Develop 2019; 23(2): 111–129.

[67] Rupe ER Rodean J Hurley EA , et al. Menstrual health among adolescents and young adults in rural Haiti. Reprod Health 2022; 19(1): 227. 36539795 10.1186/s12978-022-01533-4 PMC9764460

[68] Khan SM Bain RES Lunze K , et al. Optimizing household survey methods to monitor the Sustainable Development Goals targets 6.1 and 6.2 on drinking water, sanitation and hygiene: a mixed-methods field-test in Belize. PLoS One 2017; 12(12): e0189089. 10.1371/journal.pone.0189089 PMC5720699 29216244

[69] Santos IS Minten GC Valle NCJ , et al. Menstrual bleeding patterns: a community-based cross-sectional study among women aged 18–45 years in Southern Brazil. BMC Womens Health 2011; 11: 26. 21649903 10.1186/1472-6874-11-26 PMC3118185

[70] do Amaral MCE Hardy E Hebling EM , et al. Menstruation and amenorrhea: opinion of Brazilian women. Contraception 2005; 72(2): 157–161. 16022856 10.1016/j.contraception.2005.02.013

[71] Makuch MY Osis MJD Petta CA , et al. Menstrual bleeding: perspective of Brazilian women. Contraception 2011; 84(6): 622–627. 22078192 10.1016/j.contraception.2011.03.010

[72] Makuch MY Duarte-Osis MJ de Pádua KS , et al. Opinion and experience of Brazilian women regarding menstrual bleeding and use of combined oral contraceptives. Int J Gynecol Obstet 2012; 117(1): 5–9. 10.1016/j.ijgo.2011.11.018 22285856

[73] Marván M Cortés-Iniestra S González R. Beliefs about and attitudes toward menstruation among young and middle-aged Mexicans. Sex Role 2005; 53(3–4): 273–279.

[74] Marván ML Trujillo P. Menstrual socialization, beliefs, and attitudes concerning menstruation in rural and urban Mexican women. Health Care Women Int 2009; 31(1): 53–67. 10.1080/07399330902833362 20390636

[75] Marván ML Lama C. Attitudes toward menstrual suppression and conformity to feminine norms in young and middle-aged Mexican women. J Psychosom Obstet Gynaecol 2009; 30(3): 147–155. 19591053 10.1080/01674820903049843

[76] Szarewski A von Stenglin A Rybowski S. Women’s attitudes towards monthly bleeding: results of a global population-based survey. Euro J Contracep Reprod Health Care 2012; 17(4): 270–283. 10.3109/13625187.2012.684811 22758651

[77] Marván ML Vázquez-Toboada R Chrisler JC. Ambivalent sexism, attitudes towards menstruation and menstrual cycle-related symptoms. Int J Psychol 2014; 49(4): 280–287. 24990639 10.1002/ijop.12028

[78] Snow R Hardy E Kneuper E , et al. Women’s responses to menses and nonbleeding intervals in the USA, Brazil and Germany. Contraception 2007; 76(1): 23–29. 17586132 10.1016/j.contraception.2007.03.008

[79] Alvarado G Rivera R Ruiz R , et al. The characteristics of the menstrual bleeding pattern in a group of normal women from Durango [Caracteristicas del patron de sangrado menstrual en un grupo de mujeres normales de Durango]. Ginecol Obstet Mex 1988; 56: 127–131. 3154226

[80] Morales-Carmona F Pimentel-Nieto D Bustos-López H. Percepción del ciclo menstrual y malestar psicológico en una muestra de mujeres mexicanas. Rev Invest Clín 2008; 60(6): 478–485. 19378834

[81] Barbosa-Silva J Avila MA de Oliveira RF , et al. Prevalence, pain intensity and symptoms associated with primary dysmenorrhea: a cross-sectional study. BMC Womens Health 2024; 24(1): 92. 38311716 10.1186/s12905-023-02878-z PMC10840141

[82] Cândido JLL Maia A Cunha GMN , et al. Use of anti-inflammatory agents by pharmacy college students: correlation of the menstrual cycle and self-medication. J Young Pharm 2018; 10(4): 466–470.

[83] dos Santos LB Ferreira CWS Goncalves CG , et al. Association among dysmenorrhea and activity limitation and participation restrictions in adult women: a cross-sectional study, Brazil-2017. Arch Public Health 2021; 79(1): 194. 34753491 10.1186/s13690-021-00721-1 PMC8579669

[84] dos Santos LB Barbosa IR Dantas THD , et al. Prevalence of primary dysmenorrhea and associated factors in adult women. Rev Assoc Med Bras (1992) 2022; 68(1): 31–36. 35239934 10.1590/1806-9282.20210341

[85] Huerta-Franco MR Malacara JM. Association of physical and emotional symptoms with the menstrual cycle and life-style. J Reprod Med 1993; 38(6): 448–454. 8331624

[86] Leite RMC Buoncompagno EM Leite AC , et al. Psychosexual characteristics of female university students in Brazil. Adolescence 1994; 29(114): 439–460. 8085494

[87] Longo da Silva CM Gigante DP Carret MLV , et al. Population study of premenstrual syndrome. Rev Saude Publica 2006; 40(1): 47–56. 16410982 10.1590/s0034-89102006000100009

[88] Longo da Silva CM Gigante DP Minten GC. Premenstrual symptoms and syndrome according to age at menarche in a 1982 birth cohort in southern Brazil. Cad Saude Publica 2008; 24(4): 835–844. 18392361 10.1590/s0102-311x2008000400014

[89] Vargas-Costales JA de Las Mercedes Villa Rosero CY Mazin SC , et al. Prevalence of chronic pelvic pain and associated factors among indigenous women of reproductive age in Ecuador. BMC Womens Health 2024; 24(1): 388. 38965526 10.1186/s12905-024-03189-7 PMC11223279

[90] Victor FF Souza AI Barreiros CDT , et al. Quality of life among university students with premenstrual syndrome. Rev Bras Ginecol Obstet 2019; 41(5): 312–317. 31181584 10.1055/s-0039-1688709

[91] de Las Mercedes Villa Rosero CY Mazin SC Nogueira AA , et al. Prevalence of chronic pelvic pain and primary dysmenorrhea in women of reproductive age in Ecuador. BMC Womens Health 2022; 22(1): 363. 36056424 10.1186/s12905-022-01948-y PMC9438184

[92] Ishikura IA da Silva Vallim JR Fernandes GL , et al. Altered sleep and diurnal consequences in women with dysmenorrhea: study from the EPISONO database. Arch Gynecol Obstet 2024; 310(3): 1659–1667. 39101962 10.1007/s00404-024-07668-y

[93] Ortiz MI Fernández-Martínez E Pérez-Hernandez N , et al. Patterns of prescription and self-medication for treating primary dysmenorrhea in a Mexican population. Proc West Pharmacol Soc 2007; 50: 165–167. 18605257

[94] Pedrón-Nuevo N Gonzalez-Unzaga LN De Celis-Carrillo R , et al. Frecuencia de la dismenorrea y síntomas asociados en mujeres de 12 a 24 años. Ginecol Obstet Méx 1998; 66: 492–494. 9951177

[95] Diaz-Vélez C Vargas-Tineo OW Segura-Muñoz DM , et al. Characteristics of the use of alternative and complementary treatment in dysmenorrhea in women of fertile age. Revista Cuerpo Médico Hospital Nacional Almanzor Aguinaga Asenjo 2021; 14(4): 506–509.

[96] Rezende APR Alvarenga FR Ramos M , et al. Prevalence of premenstrual syndrome and associated factors among academics of a University in Midwest Brazil. Rev Bras Ginecol Obstet 2022; 44(2): 133–141. 35213911 10.1055/s-0041-1741456 PMC9948150

[97] Dennerstein L Lehert P Bäckström TC , et al. The effect of premenstrual symptoms on activities of daily life. Fertil Steril 2010; 94(3): 1059–1064. 19486964 10.1016/j.fertnstert.2009.04.023

[98] Pedro AO Brandão JDP de Oliveira Silva SB , et al. Impact of age on premenstrual syndrome prevalence and severity: a population-based survey in Brazil. Int J Gynecol Obstet 2024; 168: 1221–1228. 10.1002/ijgo.15895 PMC11823361 39319606

[99] Meza-Moreno FI Pimienta-Alcaraz MJ Vázquez-Valdez MF , et al. Self-reported executive function, and not performance-based measures, strongly associates with symptoms of premenstrual syndrome/premenstrual dysphoric disorder. Salud Mental 2021; 44(2): 83–90.

[100] Marván ML Díaz-Erosa M Montesinos A. Premenstrual symptoms in Mexican women with different educational levels. J Psychol 1998; 132(5): 517–526. 9729845 10.1080/00223989809599284

[101] Marván ML Díaz Eroza MC. Sintomatologia premenstrual en mujeres de area rural y urbana. Acta Psiquiátr Psicol Am Lat 1995; 41(4): 316–321. 8762707

[102] Ishikura IA Moysés-Oliveira M Fernandes GL , et al. How do phases of the menstrual cycle affect sleep? A polysomnographic study of the EPISONO database. Sleep Breath 2024; 28(3): 1399–1407. 38315317 10.1007/s11325-024-02996-4

[103] Ishikura IA Hachul H Moysés-Oliveira M , et al. Sleep is altered during menstruation but not inflammatory parameters: results from polysomnography of EPISONO database. J Sleep Res 2024; 34: e14380. 10.1111/jsr.14380 39448073

[104] Álvarez JP. These are Latin America’s leading countries by R&D spending: Bloomberg Línea, https://www.bloomberglinea.com/english/these-are-latin-americas-leading-countries-by-rd-spending/ (2023).

[105] Kashyap V Choudhari SG. Menstrual hygiene problems and challenges faced by adolescent females in rural areas: a narrative review. Cureus 2023; 15(6): e40438. 10.7759/cureus.40438 PMC10349208 37456456

[106] Rossouw L Ross H. Understanding period poverty: socio-economic inequalities in menstrual hygiene management in eight low- and middle-income countries. Int J Environ Res Public Health 2021; 18(5): 2571. 33806590 10.3390/ijerph18052571 PMC7967348

[107] Wilson L Copp T Hickey M , et al. Women who experience heavy menstrual bleeding: prevalence and characteristics from young adulthood to midlife, Australia, 2000–2021: a longitudinal cohort survey study. Med J Australia 2025; 222(4): 191–197. 39888030 10.5694/mja2.52596

[108] McCurry J Skvarc D Evans S , et al. In pain and lonely? A longitudinal study examining the associations between menstrual pain, physical functioning and loneliness. Br J Health Psychol 2025; 30(3): e12805. 10.1111/bjhp.12805 PMC12104796 40415565

[109] Åkerman E Wängborg A Persson M , et al. Navigating menstrual stigma and norms: a qualitative study on young people’s menstrual experiences and strategies for improving menstrual health. BMC Public Health 2024; 24(1): 3401. 39690406 10.1186/s12889-024-20936-5 PMC11654398

[110] Romo LF Berenson AB. Tampon use in adolescence: differences among European American, African American and Latina women in practices, concerns, and barriers. J Pediat Adolescent Gynecol 2012; 25(5): 328–333. 10.1016/j.jpag.2012.06.001 22980411

[111] Calderón-Villarreal A. Taxing women’s bodies: the state of menstrual product taxes in the Americas. Lancet Region Health Am 2024; 29: 100637. 10.1016/j.lana.2023.100637 PMC10701444 38077619

[112] King S. Why current menstrual policies do not work. Nat Human Behav 2024; 8(11): 2072–2073. 39567731 10.1038/s41562-024-01996-4

[113] Jaafar H Ismail SY Azzeri A. Period poverty: a neglected public health issue. Korean J Family Med 2023; 44(4): 183–188. 10.4082/kjfm.22.0206 PMC10372806 37189262

[114] Mohd Tohit NF Haque M . Breaking the cycle: addressing period poverty as a critical public health challenge and its relation to sustainable development goals. Cureus 2024; 16(6): e62499. 10.7759/cureus.62499 PMC11180967 38887745

[115] Nuñez A González P Talavera GA , et al. Machismo, marianismo, and negative cognitive-emotional factors: findings from the Hispanic Community Health Study/Study of Latinos Sociocultural Ancillary Study. J Latinx Psychol 2016; 4(4): 202–217. 10.1037/lat0000050 PMC5102330 27840779

[116] Sommer M Hirsch JS Nathanson C , et al. Comfortably, safely, and without shame: defining menstrual hygiene management as a public health issue. Am J Public Health 2015; 105(7): 1302–1311. 25973831 10.2105/AJPH.2014.302525 PMC4463372

[117] Olson MM Alhelou N Kavattur PS , et al. The persistent power of stigma: a critical review of policy initiatives to break the menstrual silence and advance menstrual literacy. PLoS Glob Public Health 2022; 2(7): e0000070. 10.1371/journal.pgph.0000070 PMC10021325 36962272

[118] Evans RL Harris B Onuegbu C , et al. Systematic review of educational interventions to improve the menstrual health of young adolescent girls. BMJ Open 2022; 12(6): e057204. 10.1136/bmjopen-2021-057204 PMC9185475 35676001

[119] Kosciw JG Zongrone AD. A global school climate crisis: insights on lesbian, gay, bisexual, transgender and queer students in Latin America, 2019, https://www.glsen.org/sites/default/files/2019-12/Global-School-Climate-Crisis-Latin-America-English-2019.pdf

[120] Holst AS Jacques-Aviñó C Berenguera A , et al. Experiences of menstrual inequity and menstrual health among women and people who menstruate in the Barcelona area (Spain): a qualitative study. Reprod Health 2022; 19(1): 45. 35183195 10.1186/s12978-022-01354-5 PMC8857732

[121] American College of Obstetricians and Gynecologists. ACOG Committee Opinion No. 760: dysmenorrhea and Endometriosis in the Adolescent. Obstet Gynecol 2018; 132(6): e249-e258. 10.1097/AOG.0000000000002978 30461694

[122] Firoozi R Kafi M Salehi I , et al. The relationship between severity of premenstrual syndrome and psychiatric symptoms. Iran J Psychiatry 2012; 7(1): 36–40. 23056116 PMC3395966

[123] Santibáñez MBV Gutiérrez AMC . Significados y prácticas culturales de la menstruación en mujeres aymara del norte de Chile—Un aporte desde el género a los estudios antropológicos de la sangre menstrual. Chungara 2017; 49(1): 99–108.

