# Supplementary material for: Menstrual health among young adults in Latin America and the Caribbean: A scoping review and evidence-gap map
Source: Womens Health (Lond). 2025 Oct 31;21:17455057251379612. doi: 10.1177/17455057251379612 (PMC12579131; doi:10.1177/17455057251379612)
Supplement: sj-docx-2-whe-10.1177_17455057251379612 – Supplemental material for Menstrual health among young adults in Latin America and the Caribbean: A scoping review and evidence-gap map [file sj-docx-2-whe-10.1177_17455057251379612.docx]

| **Medline via Ovid (search strategy run on 23 October 2024, with publication year limit 2023 – current)** | | | |
| --- | --- | --- | --- |
| **PCC framework** | **#** | **Keywords and** **medical subject headings (MeSH) terms** | **Search results** |
| Population: Young adults | 1 | Universities/ or Students/ or Adolescent/ or Young Adult/ or Adult/ or Female/ or Women/ | 11592719 |
|  | 2 | limit 1 to yr=" 2023 -Current" | 518445 |
|  | 3 | ((Universit* or College* or Tertiary or "Higher education" or Campus or Undergrad* or Postgrad* or "Post-grad*") adj5 Student*).ti,ab. | 75178 |
|  | 4 | limit 3 to yr=" 2023 -Current" | 9285 |
|  | 5 | (Adolescen* or "Young Adult*" or Adult* or Female or Woman or Women).ti,ab. | 3383920 |
|  | 6 | limit 5 to yr=" 2023 -Current" | 284141 |
|  | 7 | 2 or 4 or 6 | 553632 |
| Concept: Menstrual Health Experiences | 8 | Menstruation/ or Menstrual Cycle/ or Premenstrual Syndrome/ or Dysmenorrhea/ or Endometriosis/ or Adenomyosis/ or Pelvic Pain/ or Menstruation Disturbances/ or Amenorrhea/ or Menorrhagia/ or Oligomenorrhea/ | 86285 |
|  | 9 | limit 8 to yr="2023 -Current" | 4118 |
|  | 10 | (Menstrua* or Mense* or Menarch* or "Menstrual period" or Catamenia or Premenstrua* or "Pre-menstrua*" or PMS or "Premenstrual tension" or Dysmenorrh* or Endometriosis or Adenomyosis or Endometritis or "Pelvic Pain" or "Menst* Disorders" or "Menstruation Disturbances" or Amenorrhea or Menorrhagia or Oligomenorrhea or "Menstrual pain" or "Painful menst*" or "Period pain" or "Period poverty" or "Menstrual poverty").ti,ab. | 118808 |
|  | 11 | limit 10 to yr="2023 -Current" | 8414 |
|  | 12 | 9 or 11 | 8660 |
| Context: Latin America and the Caribbean | 13 | exp Caribbean region/ or exp Central America/ or exp Latin America/ or exp South America/ or exp Mexico/ | 305118 |
|  | 14 | limit 13 to yr="2023 -Current" | 19526 |
|  | 15 | ("Latin America*" or "South America*" or "Central America*" or Caribbean or “West Indies”).ti,ab. | 63522 |
|  | 16 | limit 15 to yr="2023 -Current" | 5684 |
|  | 17 | (Belize* or Costa Rica* or Cuba* or "Domincan Republic*" or "El Salvador*" or Guatemala* or Haiti* or Hondura* or Jamaica* or Mexic* or Nicaragua* or Panama* or "Saint Lucia*" or Argentin* or Bolivia* or Brazil* or Chile* or Colombia* or Ecuador* or Paraguay* or Peru* or Uruguay* or Venezuela* or "Antigua* and Barbuda*" or Aruba* or Baham* or Barbad* or Bajans or "British Virgin Island*" or "Cayman Island*" or Guyan* or "Puerto Ric*" or "Sint Maarten*" or "Saint Martin*" or Dominica* or Grenad* or "St kitts and Nevis" or Kittitians or Nevisians or "St Vincent and the Grenadines" or Vincentians or Suriname* or "Trinidad and Tobago" or Trinidadians or Tobagonians or "Turks and Caicos Island*" or Belongers or "Virgin Island*").ti,ab. | 323065 |
|  | 18 | limit 17 to yr="2023 -Current" | 26295 |
|  | 19 | 14 or 16 or 18 | 30532 |
| Final Search | 20 | 7 and 12 and 19 | 109 |

| **ERIC via ProQuest (search strategy run on 23 October 2024, with additional limits - Date: From 01 March 2023 to 23 October 2024)** | | | |
| --- | --- | --- | --- |
| **PCC framework** | **#** | **Keywords and ERIC thesaurus terms** | **Search results** |
| Population: Young adults | S1 | MAINSUBJECT.EXACT("Universities") OR MAINSUBJECT.EXACT("Students") OR MAINSUBJECT.EXACT("Adolescents") OR MAINSUBJECT.EXACT("Young Adults") OR MAINSUBJECT.EXACT("Adults") OR MAINSUBJECT.EXACT("Females") | 3042 |
|  | S2 | noft((Universit* or College* or Tertiary or "Higher education" or Campus or Undergrad* or Postgrad* or "Post-grad*") NEAR/5 Student*) | 6300 |
|  | S3 | noft(Adolescen* or "Young Adult*" or Adult* or Female or Woman or Women) | 4543 |
|  | S4 | S1 or S2 or S3 | 3 |
| Concept: Menstrual Health Experiences | S5 | noft(Menstrua* or Mense* or Menarch* or "Menstrual period" or Catamenia or Premenstrua* or "Pre-menstrua*" or PMS or "Premenstrual tension" or Dysmenorrh* or Endometriosis or Adenomyosis or Endometritis or "Pelvic Pain" or "Menst* Disorders" or "Menstruation Disturbances" or Amenorrhea or Menorrhagia or Oligomenorrhea or "Menstrual pain" or "Painful menst*" or "Period pain" or "Period poverty" or "Menstrual poverty") | 20 |
|  | *None of the MedLine Mesh terms were available in Eric via ProQuest; the closest suggestion was ‘female’ which would simply duplicate search line 1. Keyword search was determined to be sufficient.* | | |
| Context: Latin America and the Caribbean | S6 | MAINSUBJECT.EXACT("Latin Americans") | 54 |
|  | S7 | noft("Latin America*" or "South America*" or "Central America*" or Caribbean or “West Indies”) | 188 |
|  | S8 | noft(Belize* or Costa Rica* or Cuba* or "Domincan Republic*" or "El Salvador*" or Guatemala* or Haiti* or Hondura* or Jamaica* or Mexic* or Nicaragua* or Panama* or "Saint Lucia*" or Argentin* or Bolivia* or Brazil* or Chile* or Colombia* or Ecuador* or Paraguay* or Peru* or Uruguay* or Venezuela* or "Antigua* and Barbuda*" or Aruba* or Baham* or Barbad* or Bajans or "British Virgin Island*" or "Cayman Island*" or Guyan* or "Puerto Ric*" or "Sint Maarten*" or "Saint Martin*" or Dominica* or Grenad* or "St kitts and Nevis" or Kittitians or Nevisians or "St Vincent and the Grenadines" or Vincentians or Suriname* or "Trinidad and Tobago" or Trinidadians or Tobagonians or "Turks and Caicos Island*" or Belongers or "Virgin Island*") | 773 |
|  | S9 | S6 OR S7 OR S8 | 30 |
| Final Search | S10 | S4 and S5 and S9 | 1 |

| **Latin America & Iberia Database via ProQuest (search strategy run on 23 October 2024, with additional limits - Date: From 01 March 2023 to 23 October 2024)** | | | |
| --- | --- | --- | --- |
| **PCC framework** | **#** | **Keywords and Subject terms** | **Search results** |
| Population: Young adults | S1 | MAINSUBJECT.EXACT("Colleges & universities") OR MAINSUBJECT.EXACT("Students") OR MAINSUBJECT.EXACT("Teenagers") OR MAINSUBJECT.EXACT("Young adults") OR MAINSUBJECT.EXACT("Adults") OR MAINSUBJECT.EXACT("Females") OR MAINSUBJECT.EXACT("Women") | 1119 |
|  | S2 | noft((Universit* or College* or Tertiary or "Higher education" or Campus or Undergrad* or Postgrad* or "Post-grad*") NEAR/5 Student*) | 390 |
|  | S3 | noft(Adolescen* or "Young Adult*" or Adult* or Female or Woman or Women) | 1509 |
|  | S4 | S1 or S2 or S3 | 644 |
| Concept: Menstrual Health Experiences | S5 | MAINSUBJECT.EXACT("Menstruation") OR MAINSUBJECT.EXACT("Premenstrual syndrome--PMS") OR MAINSUBJECT.EXACT("Endometriosis") OR MAINSUBJECT.EXACT("Amenorrhea") | 7 |
|  | S6 | noft(Menstrua* or Mense* or Menarch* or "Menstrual period" or Catamenia or Premenstrua* or "Pre-menstrua*" or PMS or "Premenstrual tension" or Dysmenorrh* or Endometriosis or Adenomyosis or Endometritis or "Pelvic Pain" or "Menst* Disorders" or "Menstruation Disturbances" or Amenorrhea or Menorrhagia or Oligomenorrhea or "Menstrual pain" or "Painful menst*" or "Period pain" or "Period poverty" or "Menstrual poverty") | 11 |
|  | S7 | S5 or S6 | 76 |
| Context: Latin America and the Caribbean | S8 | noft("Latin America*" or "South America*" or "Central America*" or Caribbean or “West Indies”) | 889 |
|  | S9 | noft(Belize* or Costa Rica* or Cuba* or "Domincan Republic*" or "El Salvador*" or Guatemala* or Haiti* or Hondura* or Jamaica* or Mexic* or Nicaragua* or Panama* or "Saint Lucia*" or Argentin* or Bolivia* or Brazil* or Chile* or Colombia* or Ecuador* or Paraguay* or Peru* or Uruguay* or Venezuela* or "Antigua* and Barbuda*" or Aruba* or Baham* or Barbad* or Bajans or "British Virgin Island*" or "Cayman Island*" or Guyan* or "Puerto Ric*" or "Sint Maarten*" or "Saint Martin*" or Dominica* or Grenad* or "St kitts and Nevis" or Kittitians or Nevisians or "St Vincent and the Grenadines" or Vincentians or Suriname* or "Trinidad and Tobago" or Trinidadians or Tobagonians or "Turks and Caicos Island*" or Belongers or "Virgin Island*") | 8012 |
|  | *None of the MedLine Mesh terms were available in Latin America & Iberia Database via ProQuest, so only keyword searches were used.* | | |
|  | S10 | S8 or S9 | 49 |
| Final Search | 12 | S4 and S7 and S10 | 13 |

| **Psycinfo via Ovid (search strategy run on 23 October 2024, with publication year limit 2023 – current)** | | | |
| --- | --- | --- | --- |
| **PCC framework** | **#** | **Keywords and APA thesaurus terms** | **Search results** |
| Population: Young adults | 1 | colleges/ or Students/ or human females/ | 149828 |
|  | 2 | limit 1 to yr="2023 -Current" | 7355 |
|  | 3 | ((Universit* or College* or Tertiary or "Higher education" or Campus or Undergrad* or Postgrad* or "Post-grad*") adj5 Student*).ti,ab. | 184864 |
|  | 4 | limit 3 to yr="2023 -Current" | 11304 |
|  | 5 | (Adolescen* or "Young Adult*" or Adult* or “human females” or Female or Woman or Women).ti,ab. | 1228389 |
|  | 6 | limit 5 to yr="2023 -Current" | 75839 |
|  | 7 | 2 or 4 or 6 | 86726 |
| Concept: Menstrual Health Experiences | 8 | Menstruation/ or Menstrual Cycle/ or Premenstrual Syndrome/ or Dysmenorrhea/ or Gynecological Disorders/ or Amenorrhea/ or Menstrual Disorders/ | 6781 |
|  | 9 | limit 8 to yr="2023 -Current" | 411 |
|  | 10 | (Menstrua* or Mense* or Menarch* or "Menstrual period" or Catamenia or Premenstrua* or "Pre-menstrua*" or PMS or "Premenstrual Tension" or Dysmenorrh* or Endometriosis or Adenomyosis or Endometritis or "Pelvic Pain" or "Menst* Disorders" or "Menstruation Disturbances" or “Gynecological Disorders” or Amenorrhea or Menorrhagia or Oligomenorrhea or "Menstrual pain" or "Painful menst*" or "Period pain" or "Period poverty" or "Menstrual poverty").ti,ab. | 12951 |
|  | 11 | limit 10 to yr="2023 -Current" | 744 |
|  | 12 | 9 or 11 | 793 |
| Context: Latin America and the Caribbean | 13 | "latinos/latinas"/ | 30450 |
|  | 14 | limit 13 to yr="2023 -Current" | 2495 |
|  | 15 | ("Latin America*" or "South America*" or "Central America*" or Caribbean or “West Indies” or “SPANISH AMERICANS”).ti,ab. | 15077 |
|  | 16 | limit 15 to yr="2023 -Current" | 1041 |
|  | 17 | (Belize* or Costa Rica* or Cuba* or "Domincan Republic*" or "El Salvador*" or Guatemala* or Haiti* or Hondura* or Jamaica* or Mexic* or Nicaragua* or Panama* or "Saint Lucia*" or Argentin* or Bolivia* or Brazil* or Chile* or Colombia* or Ecuador* or Paraguay* or Peru* or Uruguay* or Venezuela* or "Antigua* and Barbuda*" or Aruba* or Baham* or Barbad* or Bajans or "British Virgin Island*" or "Cayman Island*" or Guyan* or "Puerto Ric*" or "Sint Maarten*" or "Saint Martin*" or Dominica* or Grenad* or "St kitts and Nevis" or Kittitians or Nevisians or "St Vincent and the Grenadines" or Vincentians or Suriname* or "Trinidad and Tobago" or Trinidadians or Tobagonians or "Turks and Caicos Island*" or Belongers or "Virgin Island*").ti,ab. | 80925 |
|  | 18 | limit 17 to yr="2023 -Current" | 5129 |
|  | 19 | 14 or 16 OR 18 | 7780 |
| Final Search | 20 | 7 and 12 and 19 | 10 |

| **Web of Science (search strategy run on 23 October 2024, with additional limits - Date: From 01 March 2023 to 23 October 2024)** | | | |
| --- | --- | --- | --- |
| **PCC framework** | **#** | **Keywords** | **Search results** |
| Population: Young adults | 1 | TS=((Universit* or College* or Tertiary or "Higher education" or Campus or Undergrad* or Postgrad* or "Post-grad*") NEAR/5 Student*) | 39,724 |
|  | 2 | TS=(Adolescen* or "Young Adult*" or Adult* or Female or Woman or Women) | 576,240 |
|  | 3 | 1 or 2 | 605,570 |
| Concept: Menstrual Health Experiences | 4 | TS=(Menstrua* or Mense* or Menarch* or "Menstrual period" or Catamenia or Premenstrua* or "Pre-menstrua*" or PMS or "Premenstrual tension" or Dysmenorrh* or Endometriosis or Adenomyosis or Endometritis or "Pelvic Pain" or "Menst* Disorders" or "Menstruation Disturbances" or Amenorrhea or Menorrhagia or Oligomenorrhea or "Menstrual pain" or "Painful menst*" or "Period pain" or "Period poverty" or "Menstrual poverty") | 17,834 |
| Context: Latin America and the Caribbean | 5 | TS=("Latin America*" or "South America*" or "Central America*" or Caribbean or “West Indies”) | 24,430 |
|  | 6 | TS=(Belize* or Costa Rica* or Cuba* or "Domincan Republic*" or "El Salvador*" or Guatemala* or Haiti* or Hondura* or Jamaica* or Mexic* or Nicaragua* or Panama* or "Saint Lucia*" or Argentin* or Bolivia* or Brazil* or Chile* or Colombia* or Ecuador* or Paraguay* or Peru* or Uruguay* or Venezuela* or "Antigua* and Barbuda*" or Aruba* or Baham* or Barbad* or Bajans or "British Virgin Island*" or "Cayman Island*" or Guyan* or "Puerto Ric*" or "Sint Maarten*" or "Saint Martin*" or Dominica* or Grenad* or "St kitts and Nevis" or Kittitians or Nevisians or "St Vincent and the Grenadines" or Vincentians or Suriname* or "Trinidad and Tobago" or Trinidadians or Tobagonians or "Turks and Caicos Island*" or Belongers or "Virgin Island*") | 115,230 |
|  | 7 | 5 or 6 | 127,911 |
| Final search | 8 | 3 and 4 and 7 | 145 |

| **Scopus (search strategy run on 23 October 2024)** | | | |
| --- | --- | --- | --- |
| **PCC framework** | **#** | **Keywords** | **Search results** |
| Population: Young adults | 1 | TITLE-ABS-KEY ( student* W/5 ( universit* OR college* OR tertiary OR "higher education" OR campus OR undergrad* OR postgrad* OR "post-grad*" ) ) AND PUBYEAR > 2022 AND PUBYEAR < 2026 | 64,721 |
|  | 2 | TITLE-ABS-KEY ( adolescen* OR "young adult*" OR adult* OR female OR woman OR women ) AND PUBYEAR > 2022 AND PUBYEAR < 2026 | 1,318,065 |
|  | 3 | TITLE-ABS-KEY ( student* W/5 ( universit* OR college* OR tertiary OR "higher education" OR campus OR undergrad* OR postgrad* OR "post-grad*" ) ) AND PUBYEAR > 2022 AND PUBYEAR < 2026 ) OR ( TITLE-ABS-KEY ( adolescen* OR "young adult*" OR adult* OR female OR woman OR women ) AND PUBYEAR > 2022 AND PUBYEAR < 2026 ) | 1,364,601 |
| Concept: Menstrual Health Experiences | 4 | TITLE-ABS-KEY ( menstrua* OR mense* OR menarch* OR "Menstrual period" OR catamenia OR premenstrua* OR "Pre-menstrua*" OR pms OR "Premenstrual tension" OR dysmenorrh* OR endometriosis OR adenomyosis OR endometritis OR "Pelvic Pain" OR "Menst* Disorders" OR "Menstruation Disturbances" OR amenorrhea OR menorrhagia OR oligomenorrhea OR "Menstrual pain" OR "Painful menst*" OR "Period pain" OR "Period poverty" OR "Menstrual poverty" ) AND PUBYEAR > 2022 AND PUBYEAR < 2026 | 26,301 |
| Context: Latin America and the Caribbean | 5 | TITLE-ABS-KEY ( "latin america*" OR "south america*" OR "central america*" OR caribbean OR “West Indies”) AND PUBYEAR > 2022 AND PUBYEAR < 2026 | 29,376 |
|  | 6 | TITLE-ABS-KEY ( belize* OR costa AND rica* OR cuba* OR "domincan republic*" OR "el salvador*" OR guatemala* OR haiti* OR hondura* OR jamaica* OR mexic* OR nicaragua* OR panama* OR "saint lucia*" OR argentin* OR bolivia* OR brazil* OR chile* OR colombia* OR ecuador* OR paraguay* OR peru* OR uruguay* OR venezuela* OR "antigua* and barbuda*" OR aruba* OR baham* OR barbad* OR bajans OR "british virgin island*" OR "cayman island*" OR guyan* OR "puerto ric*" OR "sint maarten*" OR "saint martin*" OR dominica* OR grenad* OR "st kitts and nevis" OR kittitians OR nevisians OR "st vincent and the grenadines" OR vincentians OR suriname* OR "trinidad and tobago" OR trinidadians OR tobagonians OR "turks and caicos island*" OR belongers OR "virgin island*" ) AND PUBYEAR > 2022 AND PUBYEAR < 2026 | 2,111 |
|  | 7 | ( TITLE-ABS-KEY ( "latin america*" OR "south america*" OR "central america*" OR caribbean OR "West Indies" ) AND PUBYEAR > 2022 AND PUBYEAR < 2026 ) OR ( TITLE-ABS-KEY ( belize* OR costa AND rica* OR cuba* OR "domincan republic*" OR "el salvador*" OR guatemala* OR haiti* OR hondura* OR jamaica* OR mexic* OR nicaragua* OR panama* OR "saint lucia*" OR argentin* OR bolivia* OR brazil* OR chile* OR colombia* OR ecuador* OR paraguay* OR peru* OR uruguay* OR venezuela* OR "antigua* and barbuda*" OR aruba* OR baham* OR barbad* OR bajans OR "british virgin island*" OR "cayman island*" OR guyan* OR "puerto ric*" OR "sint maarten*" OR "saint martin*" OR dominica* OR grenad* OR "st kitts and nevis" OR kittitians OR nevisians OR "st vincent and the grenadines" OR vincentians OR suriname* OR "trinidad and tobago" OR trinidadians OR tobagonians OR "turks and caicos island*" OR belongers OR "virgin island*" ) AND PUBYEAR > 2022 AND PUBYEAR < 2026 ) | 30,897 |
| Final search | 8 | ( ( TITLE-ABS-KEY ( student* W/5 ( universit* OR college* OR tertiary OR "higher education" OR campus OR undergrad* OR postgrad* OR "post-grad*" ) ) AND PUBYEAR > 2022 AND PUBYEAR < 2026 ) OR ( TITLE-ABS-KEY ( adolescen* OR "young adult*" OR adult* OR female OR woman OR women ) AND PUBYEAR > 2022 AND PUBYEAR < 2026 ) ) AND ( TITLE-ABS-KEY ( menstrua* OR mense* OR menarch* OR "Menstrual period" OR catamenia OR premenstrua* OR "Pre-menstrua*" OR pms OR "Premenstrual tension" OR dysmenorrh* OR endometriosis OR adenomyosis OR endometritis OR "Pelvic Pain" OR "Menst* Disorders" OR "Menstruation Disturbances" OR amenorrhea OR menorrhagia OR oligomenorrhea OR "Menstrual pain" OR "Painful menst*" OR "Period pain" OR "Period poverty" OR "Menstrual poverty" ) AND PUBYEAR > 2022 AND PUBYEAR < 2026 ) AND ( ( TITLE-ABS-KEY ( "latin america*" OR "south america*" OR "central america*" OR caribbean OR "West Indies" ) AND PUBYEAR > 2022 AND PUBYEAR < 2026 ) OR ( TITLE-ABS-KEY ( belize* OR costa AND rica* OR cuba* OR "domincan republic*" OR "el salvador*" OR guatemala* OR haiti* OR hondura* OR jamaica* OR mexic* OR nicaragua* OR panama* OR "saint lucia*" OR argentin* OR bolivia* OR brazil* OR chile* OR colombia* OR ecuador* OR paraguay* OR peru* OR uruguay* OR venezuela* OR "antigua* and barbuda*" OR aruba* OR baham* OR barbad* OR bajans OR "british virgin island*" OR "cayman island*" OR guyan* OR "puerto ric*" OR "sint maarten*" OR "saint martin*" OR dominica* OR grenad* OR "st kitts and nevis" OR kittitians OR nevisians OR "st vincent and the grenadines" OR vincentians OR suriname* OR "trinidad and tobago" OR trinidadians OR tobagonians OR "turks and caicos island*" OR belongers OR "virgin island*" ) AND PUBYEAR > 2022 AND PUBYEAR < 2026 ) ) | 37 |
