# Supplementary material for: Menstrual health among young adults in Latin America and the Caribbean: A scoping review and evidence-gap map
Source: Womens Health (Lond). 2025 Oct 31;21:17455057251379612. doi: 10.1177/17455057251379612 (PMC12579131; doi:10.1177/17455057251379612)
Supplement: sj-docx-3-whe-10.1177_17455057251379612 – Supplemental material for Menstrual health among young adults in Latin America and the Caribbean: A scoping review and evidence-gap map [file sj-docx-3-whe-10.1177_17455057251379612.docx]

Supplementary Material 3. Inclusion and exclusion criteria

| **Criteria** | **Inclusion** | **Exclusion** |
| --- | --- | --- |
| **General criteria** | Publication time scan (01 January 1980- 23 October 2024); English, Spanish, Portuguese or French language; Primary studies (original research) with qualitative and/or quantitative study design (e.g., descriptive) | Publication time scan (before 1980); Secondary studies (i.e. reviews); Validation only studies; Experimental studies; Abstracts where the full study is not reported e.g., Conference abstract |
| **Population** | Young adults who menstruate within the age ranges of 18–24-years including transgender individuals and gender diverse individuals in university, healthcare or broader community settings (including workplaces, households, and other non-specific community-based studies) | Adolescents or older adults outside of the specified age range; Studies conducted in secondary schools (where data cannot be extrapolated to outside of these settings); airline stewardesses; sex workers; incarcerated individuals; individuals in detention; Refugees; Migrants travelling through the border or currently in informal settlements; individuals with disabilities; individuals with affective disorders; individuals with Fibromyalgia; experiences of MH as reported by male partners or physicians (gynecologists) caring for reproductive-age individuals; Elite athletes; Smaller minority groups (minority religion or remote indigenous communities) |
| **Concept** | Studies involving any experience of MH, including broader sexual and reproductive health or Water Sanitation and Hygiene (WASH) studies if any component surrounds MH, this includes experiences of menstruation or menstrual related symptoms, such as eumenorrhea (normal menstrual bleeding) including bleeding patterns, or menstrual cycle length i.e. frequency of bleeding length (2-7 days) occurring every 21 to 35 days, disorders: primary dysmenorrhea and mild – moderate premenstrual syndrome/disorder, or attitudes towards menstruation, including views to suppress menstruation or induce amenorrhea using hormonal contraception; management of menstrual-related symptoms, included using non-steroidal inflammatory drugs to address pain;  Studies exploring the impact of MH on academic activities such as absenteeism or general academic performance, concentration, ability to complete tasks, productivity, and daily life included a combination of partner (such as interference with sexual intercourse) or family relationships, domestic, functional, professional and social activities (including participating in sport), quality of life, or daily life in general | Experiences of menarche; endometriosis, polycystic ovarian syndrome, chronic pelvic pain (including non-cyclic pelvic pain and dyspareunia), severe premenstrual syndrome/disorder or premenstrual dysphoric disorder, abnormal uterine bleeding, menorrhagia (heavy bleeding and/or bleeding for 7+days), specific focus of amenorrhea, irregular or short/long cycles in healthy weight or obese individuals, bacterial vaginosis, dyspareunia; focus on menstruation and fertility; Measurement of menstrual blood loss and body iron stores; Changes of food intake/food cravings during menstrual cycle; Evaluation studies evaluating interventions or products to improve MH i.e. modern disposable pads vs ‘older’ disposable pads or hysterectomy, evaluating hormonal contraception and the impact on menstrual bleeding or MH in general; evaluating frequency and experience in the use of menstrual cycle monitoring applications (apps); Experimental (including randomized control trials) to improve MH i.e. Naproxen, paracetamol and pamabrom versus paracetamol, pyrilamine and pamabrom to treat dysmenorrhea or cryotherapy to relieve dysmenorrhea; or studies involving designing MH solutions i.e. Educational resources; Studies concerned about menstruation alterations after COVID-19 vaccinations; Studies focusing solely on the impact of MH on sexual function (i.e. Impact of PMS on sexual function) or sleep quality including insomnia and fatigue (i.e. Impact of PMS on sleep quality) and not other daily life outcomes; Studies focusing solely on the effects of MH on personality; Studies focusing on exercise performance at high-altitude; Studies on hormone profiles during the menstrual cycle |
| **Context** | Countries in the Latin American and Caribbean region, as defined by the World Bank Group | Studies not conducted in the Latin American and Caribbean region, including countries from North America (e.g., the United States and Canada), Europe, Asia, Africa, and Oceania. |
